# Spatial Transcriptomic and Metabolomic Landscapes of Oral Submucous Fibrosis‐Derived Oral Squamous Cell Carcinoma and its Tumor Microenvironment

**DOI:** 10.1002/advs.202306515

**Published:** 2024-01-16

**Authors:** Yuan Zhi, Qian Wang, Moxin Zi, Shanshan Zhang, Junshang Ge, Keyue Liu, Linsong Lu, Chunmei Fan, Qijia Yan, Lei Shi, Pan Chen, Songqing Fan, Qianjin Liao, Can Guo, Fuyan Wang, Zhaojian Gong, Wei Xiong, Zhaoyang Zeng

**Affiliations:** ^1^ Department of Oral and Maxillofacial Surgery The Second Xiangya Hospital of Central South University Changsha Hunan 410011 China; ^2^ NHC Key Laboratory of Carcinogenesis and Hunan Key Laboratory of Cancer Metabolism Hunan Cancer Hospital and the Affiliated Cancer Hospital of Xiangya School of Medicine Central South University Changsha Hunan 410078 China; ^3^ Key Laboratory of Carcinogenesis and Cancer Invasion of the Chinese Ministry of Education Cancer Research Institute and School of Basic Medicine Sciences Central South University Changsha Hunan 410078 China; ^4^ Department of Stomatology Xiangya Hospital Central South University Changsha Hunan 410008 China

**Keywords:** oral squamous cell carcinoma (OSCC), oral submucous fibrosis (OSF), polyamine metabolism, spatial metabolomics, spatial transcriptomics, tumor microenvironment

## Abstract

In South and Southeast Asia, the habit of chewing betel nuts is prevalent, which leads to oral submucous fibrosis (OSF). OSF is a well‐established precancerous lesion, and a portion of OSF cases eventually progress to oral squamous cell carcinoma (OSCC). However, the specific molecular mechanisms underlying the malignant transformation of OSCC from OSF are poorly understood. In this study, the leading‐edge techniques of Spatial Transcriptomics (ST) and Spatial Metabolomics (SM) are integrated to obtain spatial location information of cancer cells, fibroblasts, and immune cells, as well as the transcriptomic and metabolomic landscapes in OSF‐derived OSCC tissues. This work reveals for the first time that some OSF‐derived OSCC cells undergo partial epithelial–mesenchymal transition (pEMT) within the in situ carcinoma (ISC) region, eventually acquiring fibroblast‐like phenotypes and participating in collagen deposition. Complex interactions among epithelial cells, fibroblasts, and immune cells in the tumor microenvironment are demonstrated. Most importantly, significant metabolic reprogramming in OSF‐derived OSCC, including abnormal polyamine metabolism, potentially playing a pivotal role in promoting tumorigenesis and immune evasion is discovered. The ST and SM data in this study shed new light on deciphering the mechanisms of OSF‐derived OSCC. The work also offers invaluable clues for the prevention and treatment of OSCC.

## Graphical abstract

Betel nut chewing is linked to the development of OSF and, in severe cases, OSCC. Employing spatial multi‐omics technology, this study unveils the malignant progression of OSCC cells, intricate cell‐to‐cell interactions, and the identification of abnormal polyamine metabolism in OSF‐derived OSCC. These findings offer novel insights into the underlying mechanisms of OSCC.

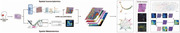

## 1 Introduction

Oral squamous cell carcinoma (OSCC) is one of the most common head and neck cancers. In 2020, there were over 370,000 diagnosed cases of OSCC worldwide, with over 170,000 deaths caused by this disease.^[^ ^1^ ^]^ More than 30% of these cases occurred in South and Southeast Asia, where betel nut chewing is a significant risk factor contributing to the high incidence of OSCC.^[^ ^2^, ^3^ ^]^ In China, provinces such as Hunan and Hainan have a considerably big population, especially males, with a long‐standing habit of chewing betel nuts, resulting in a high prevalence of OSCC.^[^ ^4^ ^]^ For example, the incidence of OSCC in males in Hunan province ranks fifth among all malignant tumors, next only to lung, colorectal, liver, and gastric cancers, and surpassing nasopharyngeal carcinoma, esophageal cancer, leukemia, and other malignancies.^[^ ^5^ ^]^

Prolonged betel nut chewing can lead to oral submucous fibrosis (OSF), clinically characterized by an extensive white fibrous network with scar‐like formations, accompanied by a burning sensation of the oral mucosa, progressive restriction of mouth opening, and trismus.^[^ ^6^, ^7^ ^]^ Pathologically, OSF is characterized by epithelial cell atrophy, abnormal proliferation, and activation of fibroblasts with excessive collagen synthesis, as well as infiltration of inflammatory cells.^[^ ^8^, ^9^ ^]^ Furthermore, OSF is a well‐established precancerous lesion, and some cases of OSF eventually progress to OSCC.^[^ ^10^, ^11^ ^]^ This process essentially involves long‐term chronic nonresolving inflammation which induces malignant transformation of epithelial cells, leading to the acquisition of various malignant biological phenotypes, including proliferation, invasion, migration, and immune evasion.^[^ ^12^, ^13^ ^]^ However, the key molecular events involved in this process are still unknown, and the complex interactions between tumor cells, fibroblasts, and immune cells in OSCC tissues remain poorly defined. The potential impact of metabolic reprogramming and metabolite change in betel nut chewing‐caused OSCC on the tumor microenvironment has not been reported.

Single‐cell RNA‐sequencing (scRNA‐seq) technique provides a transcriptomic atlas at high resolution for different cell types within tumor tissues, enabling us to explore the interactive patterns between various cells in the tumor microenvironment.^[^ ^14^, ^15^ ^]^ However, due to the mechanical dissociation and enzymatic digestion required to apportion tumor tissues into single‐cell suspensions for scRNA‐seq,^[^ ^16^ ^]^ the information about spatial relationships between cells within pathological tissues is lost. As a result, the original interaction information between neighboring cells in the tumor microenvironment, such as the expression of various cell membrane ligand–receptor pairs on adjacent cells, cannot be obtained. The emergence of Spatial Transcriptomics (ST) overcomes these limitations by directly obtaining transcriptomic expression profiles at a resolution close to the single‐cell level from frozen tissue sections while preserving the spatial location information of each cell on the tissue section.^[^ ^17^, ^18^, ^19^ ^]^ This addresses the limitations of scRNA‐seq in reflecting the spatial distribution of different cells and their related positional information within the tumor microenvironment.^[^ ^20^ ^]^ Using scRNA‐seq data to assist in annotating ST data can further enhance the sensitivity of the analysis.^[^ ^21^, ^22^ ^]^ On the other hand, the progress of mass spectrometry imaging (MSI) has facilitated the development of Spatial Metabolomics (SM). The improved airflow‐assisted desorption electrospray ionization–mass spectrometry imaging (AFADESI–MSI) technique improves the in situ capture and transfer efficiency of droplets, enhancing the high‐throughput identification of small‐molecule metabolites in tissue sections at a micron resolution while preserving their spatial location information on the tissue section, revealing the SM of tumor cells and their microenvironment near single‐cell resolution.^[^ ^23^ ^]^

**Figure 1:**
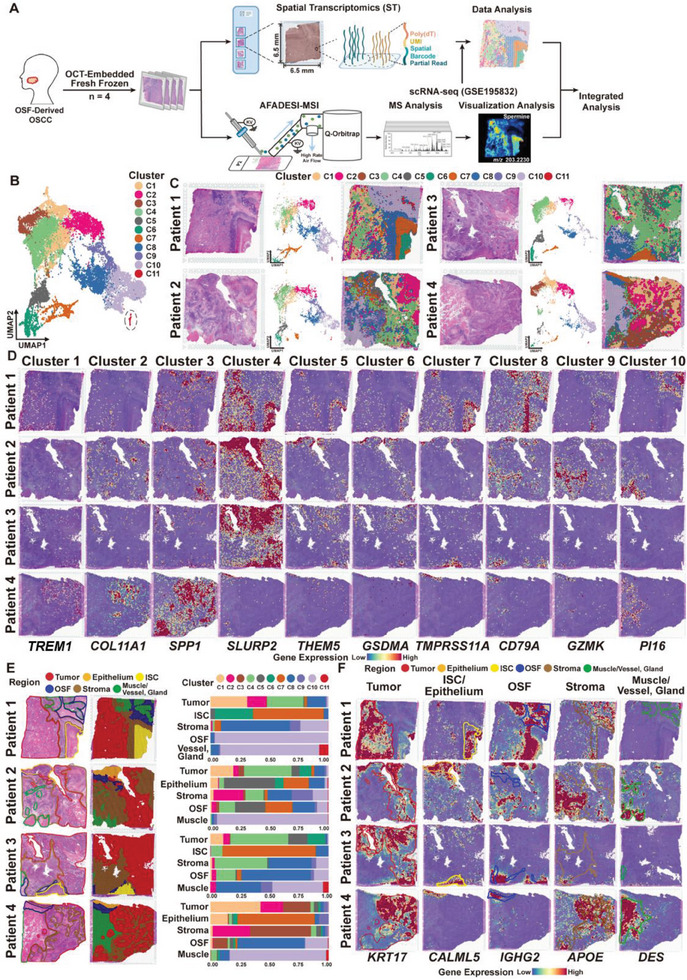
ST Landscape of OSF‐derived OSCC. A) Schematic diagram exhibiting the detection and analysis of ST and SM in the OSF‐derived OSCC. B) Unsupervised clustering analysis UMAP divided 17,319 spots from four OSF‐derived OSCC samples into 11 clusters. Cluster 11 (Marked by a dotted circle) was excluded from further analysis due to the limited number of spots (only 77). C) H&E staining images (left), UMAP plots (middle), and ST feature plots (right) of 11 clusters in four OSF‐derived OSCC samples. D) ST feature plots showing the spatial distribution of representative marker genes of each cluster in four samples. E) The tissue of four samples was divided into five regions based on the histopathological features, including tumor, adjacent epithelium, ISC, OSF, stroma, and muscle/vessel/gland regions. H&E staining images (left), ST images of spots with tissue regions annotated by different colors (middle), and the proportion of spots and corresponding clusters in each tissue region (right) were presented, respectively. F) ST feature plots showing the expression of representative marker genes in each spot of different tissue regions.

In this study, we integrate ST and SM techniques to obtain the ST and SM landscapes of OSCC tissues derived from OSF (**Figure** **1**A). We deciphered the transcriptomic and metabolomic profiles of tumor cells, fibroblasts, and immune cells within the OSCC tissues; discovered a malignant progression process from in situ carcinoma (ISC) to partial epithelial–mesenchymal transition (pEMT),^[^ ^24^ ^]^ and finally acquired a cancer‐associated fibroblast (CAF)‐like phenotype in OSCC tissues. We explored the patterns of immune cell infiltration and their potential regulations and interactions with the tumor microenvironment. Importantly, we unveiled major metabolic reprogramming in OSCC, with the most significant changes observed in polyamine metabolism. ST data revealed abnormal expression of several key enzymes involved in polyamine metabolism, driving the metabolic reprogramming in OSCC cells, which may in turn remodel the tumor microenvironment.

## 2 Results and Discussion

### 2.1 The ST Profiles of OSCC Reveal Intratumor Heterogeneity

To investigate the spatial multi‐omics characteristics of OSF‐derived OSCC and decipher key molecular events during the malignant transformation process, we collected fresh tissue samples from four clinically diagnosed OSCC patients with OSF (Table S1, Supporting Information) and prepared frozen sections for hematoxylin and eosin (H&E) staining. Considering the maximum tissue section size (6.5 × 6.5 mm) that could be detected by the ST and SM platforms, representative areas of each sample containing tumor, adjacent epithelium, OSF, and stromal tissues were selected, and two consecutive slices adjacently were used for ST and SM analysis, respectively. H&E staining showed that collagen deposition and lymphocyte infiltration were found in the subepithelial layer of adjacent epithelium in each sample, which was consistent with the clinical characteristics of OSF. In Patient 1 (P1) and Patient 3 (P3), *Z*ISC was observed in the Adjacent Epithelium, while in Patient 2 (P2) and Patient 4 (P4), epithelial dysplasia was observed in the Adjacent Epithelium. Compared to other samples, P4 exhibited a poorer differentiation of tumor cells and a higher distribution of stroma cells in Tumor regions (Figure S1A, Supporting Information). Meanwhile, we also performed Masson's staining on the above four samples and used one OSCC sample (male, 66 years old, smoking for 50 years with 2.5 packs per day, drinking alcohol for 47 years with 250 mL per day, never chewing betel nut, pT3N1M0 with clinical stage III) with no history of betel nut chewing and no OSF phenotype as a control. The results showed that significant deposition of collagen fibers was seen in the OSF regions in all OSF‐derived OSCC samples, whereas no collagen deposition was seen underneath the tumor and adjacent epithelial tissues in the control sample (Figure S1B, Supporting Information).

We performed ST and SM analysis on these four samples simultaneously. The schematic workflow of sample collection and spatial multi‐omics detection is shown in Figure 1A. By using the 10× Genomics Visium platform for ST (Figure S2A, Supporting Information), a total of 17,319 spots were detected from the four OSF‐derived OSCC samples, with an average of 12,601 unique molecular identifiers (UMIs) representing 3,056 genes per spot (Figure S2B, Table S2, Supporting Information). After integrating and normalizing spots from each sample, uniform manifold approximation and projection (UMAP),^[^ ^25^ ^]^ a nonlinear dimensionality reduction algorithm, was performed for unsupervised clustering analysis, confirming the absence of obvious batch effects among samples (Figure S2C, Supporting Information). All 17,319 spots from four samples were partitioned into 11 clusters (Figure 1B). Based on the spatial distribution of each spot on the tissue section, Clusters 1 to 4 (C1–C4), mainly located in the Tumor regions, were categorized as Tumor subtypes, Clusters 5 to 7 (C5–C7), located in the Adjacent Epithelium regions, were categorized as Adjacent Epithelium subtype. Cluster 8 (C8) and Cluster 9 (C9) in the regions with infiltrated lymphocytes were recognized as Immune subtype; Cluster 10 (C10) and Cluster 11 (C11), located in the Stroma regions enriched with collagen, muscle fibers, and blood vessels, were identified as Stroma subtypes. Due to the predominant location (muscle/vessel/gland region) and the limited spots (only 77 spots), C11 was excluded from subsequent analysis (Figure 1C; Figure S2D, Supporting Information).

Next, we annotated the biological features of each cluster based on the top ten marker genes that were significantly differentially expressed, revealing transcriptional similarities among the subtypes (Figure S2E, Supporting Information). In detail, triggering receptors expressed on myeloid cells‐1 (*TREM1*) and neurofilament light polypeptide (*NEFL*), specific marker genes of the tumor microenvironment, were significantly expressed in the C1 of OSF‐derived OSCC (Table S1, Supporting Information).^[^ ^26^ ^]^ C2 showed the specific expression of fibroblast‐related genes (i.e., *COL11A1*, *ADAM12*, *ITGA11*), suggesting a potential phenotype of epithelial–mesenchymal transition (EMT) in C2. The stroma‐related genes (i.e., *SPP1*, *MMP11*, *MMP28*, *CST1*) were highly expressed in the C3 predominantly contributed by P4, the Tumor region of which exhibited a poor degree of tumor differentiation. For instance, the extracellular matrix‐associated gene *SPP1* (encoding osteopontin) was specifically expressed in C3, which was involved in the infiltration of macrophages and the formation of immunosuppressive microenvironment.^[^ ^27^ ^]^ Marker genes of C4 are primarily associated with the biofunctions of epithelial cell differentiation (i.e., *SLURP2*, *DSG1*, *S100A7*, *TGM1*). Intriguingly, these differentiation‐related marker genes including *THEM5* and *GSDMA*, were also significantly expressed in the Adjacent Epithelium subtype (i.e., C5 and C6) (Figure S2E, Supporting Information). C7 of the Adjacent Epithelium subtype still retained the expression of marker genes represented as the signatures of normal epithelial cells (i.e., *TMPRSS11A*, *KRT3*, *GSTA1*). In the Immune subtype, B cell‐specific marker genes (i.e., *CD79A*, *PIM2*, *POU2AF1*) and T cell‐specific marker genes (i.e., *GZMK*, *CD8B*, *GZMM*) were prominently expressed in C8 and C9, respectively. The Stroma subtype (C10) exhibited significant expression of genes of myocytes (i.e., *XIRP2*, *SMPX*, *MYOT*, *LMOD2/3*, *SMTNL2*), fibroblasts (i.e., *PI16*), as well as vascular endothelial cells and erythrocytes (i.e., *SPTB*, *ATP1A2*, *AGT*) (Figure 1D; Figure S2E, Supporting Information).

To validate the correlation between the transcriptional features and the histopathological characteristics in these four samples, all the spots were separated into region types in accordance with their tissue distributions, including Tumor, Adjacent Epithelium, ISC, OSF, Stroma, and Muscle/Vessel/Gland regions. The results showed that clusters constitution (Figure 1E) and transcriptomic patterns (Figure 1F) were consistent with the pathological features of each tissue region, such as epithelial cell marker *KRT17* in the Tumor region,^[^ ^28^ ^]^ differentiation‐related gene *CALML5* in the Adjacent Epithelium or ISC region,^[^ ^29^ ^]^ immune‐related gene *IGHG2* in the OSF region,^[^ ^30^ ^]^ lipid metabolism‐related apolipoprotein *APOE* in the Stroma region,^[^ ^31^, ^32^ ^]^ and myocyte signature *DES* in the Muscle region (Figure 1F).^[^ ^33^ ^]^

In summary, we generated the ST profile of OSCC tissues with OSF induced by long‐term betel nut chewing and its evolutionary dynamics for the first time. This profile encompasses representative tissues of Adjacent Epithelium, ISC, OSF, and Stroma tissues. Meanwhile, 11 distinct clusters were identified from the ST data of OSF‐derived OSCC samples. By integrating transcriptomic data with spatial information and corresponding histopathological features, we have laid the foundation for further analysis of cell type diversity, cellular evolution, and intercellular communication within OSCC originating from OSF.

### 2.2 Spatio‐Temporal Evolution Model of ISC‐pEMT‐CAF in OSCC

We analyzed the spatio‐temporal evolution model based on the transcriptional profile of epithelial cells during the malignant progression from OSF to OSCC. Considering the limited spatial resolution of ST (the resolution of the 10× Genomics Visium platform is 55 × 55 µm, with an average of 2–30 cells per spot), we integrated published scRNA‐seq data of OSCC (GSE195832)^[^ ^34^ ^]^ and performed *SPOTlight* analysis^[^ ^35^ ^]^ on each spot to calculate the proportion and distribution of epithelial cells on Tumor, ISC, and Adjacent Epithelium regions (Figure S3A, Supporting Information). First, we re‐annotated the cell types on the GSE195832 data, and obtained six cell types, including epithelial cells, endothelial cells, fibroblasts, lymphocytes, myeloid cells, and muscle cells, with their characteristic gene expression signature. Then, we deconvoluted six cell types in each spot in our ST data and found that the spots in the malignant epithelial regions (i.e., Tumor and Adjacent Epithelium regions) were mainly composed of epithelial cells. These spots also exhibited infiltration of myeloid cells, as well as varying proportions of lymphocytes, fibroblasts, and muscle cells, reflecting the pathological features of active inflammatory response and enhanced collagen deposition in the malignant transformation of OSF‐derived OSCC (Figure S3B, Supporting Information). Meanwhile, we calculated the proportions of epithelial cells in different spots corresponding to the clusters (Figure 1B) and found that epithelial cells were predominantly presented in C1–C7 of malignant epithelial regions (Figure S3C, Supporting Information).

**Figure 2:**
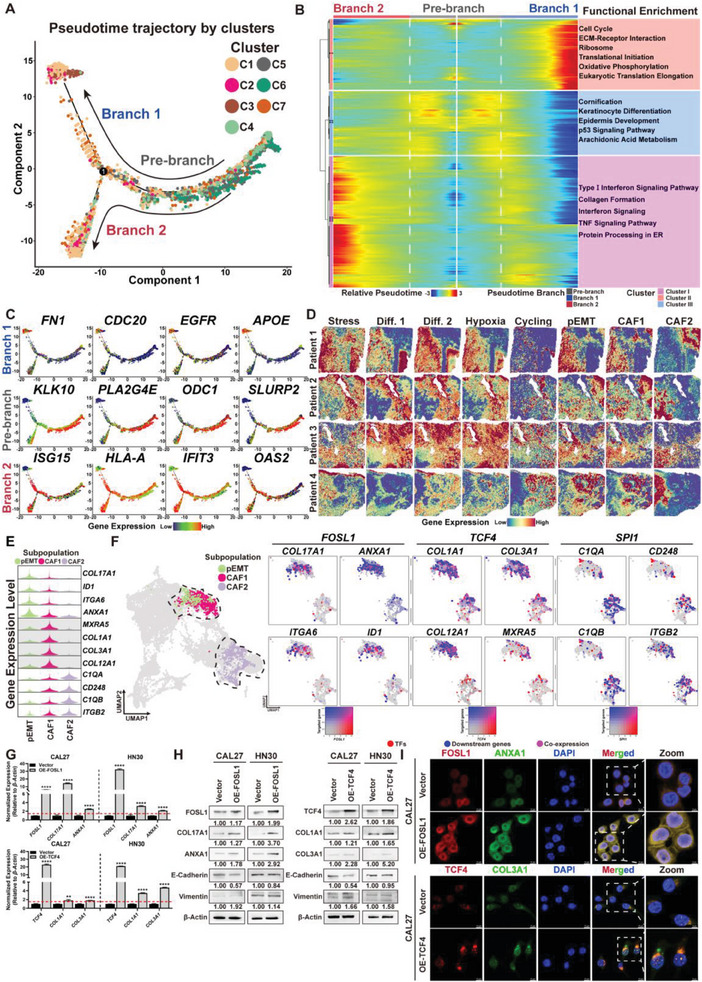
Pseudotime Analysis Revealing the ISC‐pEMT‐CAF‐like Phenotype Evolution Pattern in OSF‐derived OSCC. A) Trajectory reconstruction of epithelial cells in the malignant epithelial tissues of OSF‐derived OSCC consisted of three branches: pre‐branch (before bifurcation), T1 branch (top), and T2 branch (bottom). Each point corresponds to a spot. B) BEAM heatmap plot displaying the expression patterns of pseudotime‐specific genes and the corresponding GO pathway terms (hierarchically clustered into three profiles) in the malignant trajectory. C) The dynamic expression of pseudotime‐specific genes in each branch of the malignant trajectory. D) ST feature plots of the spatial distributions of each cell subpopulation in the four samples in accordance with public HNSCC scRNA‐seq data (Puram et al.). pEMT and CAF1 subpopulations showed a closer distribution in the tissue sections. CAF1 (mainly at the Tumor region) and CAF2 (mainly at the Stroma region) had little overlapping distribution in the tissue sections. Diff. 1: differentiation subpopulation‐1 (early differentiation stage); Diff. 2: differentiation subpopulation‐2 (late differentiation stage); pEMT: partial EMT subpopulation. E) Violin plots showing the expression of representative marker genes associated with the EMT process in three cell subpopulations of ISC‐pEMT‐CAF trajectory. There are three types of marker genes (from top to bottom): genes specific in the pEMT subpopulation (*COL17A1*, *ANXA1*, *ITGA6*, *ID1*), genes specific in CAF1 (*COL1A1*, *COL3A1*, *COL12A1*, *MXRA5*), and genes specific in CAF2 (*C1QA*, *CD248*, *C1QB*, *ITGB2*). F) ST feature plots exhibiting the strategy of subpopulation extraction and the co‐expression of TF‐targeted genes. Left: the distributions of three subpopulations in the total UMAP plot; right: the co‐expression patterns of subpopulation‐specific TFs and their targeted genes in the extracted UMAP plots. G) qRT‐PCR revealing the role of FOSL1 and TCF4 in upregulating their respective downstream target genes (e.g., *COL17A1*, *ANXA1* and *COL1A1*, *COL3A1*) in OSCC cell lines CAL27 and HN30. Upper: overexpression of FOSL1 in CAL27 and HN30 upregulated *COL17A1* and *ANXA1* gene transcription. Lower: overexpression of TCF4 in CAL27 and HN30 upregulated the expression of the downstream genes *COL1A1* and *COL3A1*. *β‐Actin* served as an endogenous molecule for normalization, and each group consisted of three replicate samples. A *p‐value* of less than 0.05 was considered statistically significant. **: *p* < 0.01, ****: *p* < 0.001. H) Western blotting detecting the effects of overexpression of FOSL1 or TCF4, respectively, in OSCC cells on the translation levels of their downstream genes and EMT markers. Left: overexpression of FOSL1 resulted in upregulation of COL17A1, ANXA1, and mesenchymal marker Vimentin, and downregulation of epithelial marker E‐Cadherin. Right: overexpression of TCF4 resulted in elevated expression of COL1A1, COL3A1, and mesenchymal marker Vimentin, and decreased expression of epithelial marker E‐Cadherin. *β* ‐Actin served as the internal reference protein. The protein concentration in the Vector group was used to normalize the relative expression of the target proteins after overexpression of FOSL1 or TCF4. I) Immunofluorescence showing the expression pattern of downstream genes (FOSL1: ANXA1; TCF4: COL3A1) in CAL27 cells transfected with FOSL1 and TCF4, respectively. Red: FOSL1 or TCF4; green: downstream genes (FOSL1: ANXA1; TCF4: COL3A1); blue: DAPI‐stained nucleus. Magnification: 630× with a scale bar of 10 µm.

In order to explore the spatio‐temporal transcriptomic characteristics during the malignant transformation of OSF‐derived OSCC, we selected a total number of 5,632 spots in C1–C7 (representing tumor and adjacent epithelial cells, Figure S4A, Supporting Information) for pseudotime analysis by using *Monocle2*,^[^ ^36^ ^]^ while C8–C10 were excluded from the subsequent pseudotime analysis due to the absence of epithelial cell distribution. We constructed a malignant progression trajectory of OSCC (**Figure** **2**A), in which the initiation of OSCC (Pre‐branch) occurred in the Adjacent Epithelium or ISC region (spots from C5–C7) and C4 with remarkable differential ability. Subsequently, the trajectory diverged into two branches (Branch 1 and Branch 2) based on the difference in transcriptional profile. C1 and C2 were distributed in both Branch 1 and Branch 2, representing as the potential common features of two evolutionary directions in OSF‐derived OSCC. Moreover, C3 and C4 played determining roles that predominantly influenced the evolutionary directions of Branch 1 and Branch 2 (Figure S4B, Supporting Information).

To further explore the temporal and functional differences in the malignant trajectory of OSF‐derived OSCC, we sought to annotate the differentially expressed genes in the spots along the two branches. The pre‐branch primarily exhibited the biofunctions of epithelial cell growth, differentiation, and keratinization (e.g., *KLK10*, *KLK12*, *KRT8*, *EVPL*, *SLURP2*). Additionally, genes involved in metabolic pathways such as arachidonic acid and amino acid metabolism (e.g., *PLA2G4E*, *ALOX12B*, *ODC1*) were also observed. In Branch 1, tumor cells displayed significant enrichment of cell cycle and translation genes (e.g., *CDC20*, *EGFR*) as well as certain metabolic genes (e.g., methyltransferase *NNMT*, apolipoprotein *APOE*), while tumor cells in Branch 2 were primarily associated with interferon response (e.g., *ISG15*, *HLA‐A*, *IFIT3*, *OAS2*). Interestingly, both Branch 1 and Branch 2 exhibited prominent expression of genes related to extracellular matrix remodeling (Branch 1: ECM‐receptor interaction, e.g., *FN1*, *COL16A1*, *VIM*, *NOTCH1*; Branch 2: collagen formation, e.g., *COL17A1*, *CSPG4*), suggesting that EMT is a common terminal phenotype in OSF‐derived OSCC for both Branch 1 and Branch 2 (Figure 2B,C; Figure S4C, Supporting Information).

Previously, Puram et al.^[^ ^24^ ^]^ identified six subpopulations of tumor cells, including stress, Differentiation‐1 (Diff1), Differentiation‐2 (Diff2), hypoxia, cycling, pEMT, and two subpopulations of cancer‐associated fibroblasts (CAF1 and CAF) in head and neck squamous cell carcinoma (HNSCC) using scRNA‐seq. However, they did not further distinguish the biological functions of CAF1 and CAF2. Using the characteristic gene sets of the above eight cell subpopulations to score all spots in our ST data, we found that stress, Diff1, Diff2, and Hypoxia subpopulations were the main cell types in the early stage of malignant transformation (C5–C7, the main components of pre‐branch of pseudotime trajectory, Figure S4B,D,E, Supporting Information). Intriguingly, both pEMT and CAF1 subpopulations held a majority in the terminal stages of OSCC (C2) while CAF2 was mainly found in C10 (Stroma subtype spots, Figure S4B,D,E, Supporting Information), indicating that spots from CAF1 subpopulation had more similar gene expression pattern with pEMT rather than CAF2 (Figure S4E, Supporting Information). We further analyzed the spatial distribution of the above eight cell subpopulations on the tissue sections of OSF‐derived OSCC and found that CAF1 spots were primarily located at the Tumor region and the invasive front, which highly overlapped with the distribution of pEMT spots. Additionally, CAF2 mainly infiltrated the OSF and Stromal region (C10), neighboring the immune subtype (C8 and C9) (Figure 2D).

EMT is a dynamic process during which malignant epithelial cells acquire mesenchymal phenotypes. During this process, pEMT cells exhibit both epithelial and early‐stage mesenchymal features and are considered the leading cells of tumor invasion and migration in various tumors.^[^ ^37^, ^38^, ^39^, ^40^ ^]^ CAF1 exhibited similarities in transcriptional patterns and spatial distribution with pEMT while significant differences from CAF2, which suggested that CAF1 represented a subset of tumor cells in C2 that have undergone EMT and acquired fibroblast (mesenchymal)‐like phenotypes, while CAF2 was the CAFs in the adjacent non‐cancerous tissue induced and recruited by OSCC cells.

To further validate this hypothesis, we performed unsupervised principal component analysis (PCA) on the spots with pEMT, CAF1, and CAF2 characteristics. PCA results showed that pEMT cells were closer to CAF1 cells, while CAF2 exhibited remarkable transcriptional differences (Figure S4G, Supporting Information). Differential expression gene (DEG) analysis and Gene Ontology enrichment by biological process (GO: BP) were conducted on the three cell types. It was found that both pEMT and CAF1 cells showed significant expression of mesenchymal markers (pEMT: *PRSS23*, *COL17A1*, *SERPINE1*, *ITGA6*; CAF1: *POSTN*, *FN1*, *COL1A1*, *COL12A1*). In comparison to the activated organization of collagen fibril and extracellular matrix in CAF1, CAF2 exhibited unique DEGs associated with immune responses such as complement activation and humoral immunity (e.g., *C3*, *CXCL12*, *PLA2G2A*, *PTGDS*) (Figure S4H,I, Table S3, Supporting Information). These findings were consistent with the transcriptional features of antigen‐presenting cancer‐associated fibroblasts (apCAFs) previously identified in other tumors.^[^ ^41^, ^42^, ^43^ ^]^

To investigate the functional differences among the pEMT, CAF1, and CAF2 cell subpopulations, we applied the transcription factor (TF) analysis algorithm *SCENIC*.^[^ ^44^ ^]^ 104 differentially expressed TFs were identified in the three subpopulations (Figure S4J, Supporting Information), while classical EMT TFs (such as *Snail*, *Slug*, *Twist*) did not show differential expression. We selected the most differentially expressed TFs in each subpopulation (Figure S4K,L, Supporting Information) and their underlying downstream genes (Figure 2E,F). *FOSL1*, a highly expressed TF in the pEMT subpopulation, showed potential regulation of EMT‐related genes (e.g., *COL17A1*, *ANXA1*, *ITGA6*, *ID1*).^[^ ^45^, ^46^ ^]^ Consistently, the high expression of extracellular matrix markers (e.g., *COL1A1*, *COL3A1*, *COL12A1*, *MXRA5*) in the CAF1 subpopulation was regulated by *TCF4*,^[^ ^47^, ^48^ ^]^ suggesting that AP‐1, TCF family might be key TFs involved in regulating the EMT process and generating CAF1‐like phenotypes in OSF‐derived OSCC. However, in CAF2, immune response‐related TF *SPI1* ^[^ ^49^ ^]^ showed more capability in stimulating the immune regulation of CAF2 subpopulation via modulating the expression of genes for complement and immune checkpoint molecules (e.g., *C1QA*, *C1QB*, *CD248*, *ITGB2*) (Figure 2E,F),^[^ ^50^, ^51^, ^52^, ^53^ ^]^ suggesting that immune microenvironment could stimulate fibroblasts into immunostimulatory cells. Subsequently, to validate the regulatory effects of the aforementioned representative TFs on downstream genes and the progression of the “ISC‐pEMT‐CAF” axis, we overexpressed FOSL1 or TCF4 in OSCC cell lines CAL27 and HN30, respectively. The results of qRT‐PCR revealed a significant increase in the transcription levels of downstream genes, including COL17A1, ANXA1, COL1A1, and COL3A1 (Figure 2G), which is consistent with the ST results. Western blotting and immunofluorescence analyses demonstrated a notable upregulation of FOSL1 downstream genes (COL17A1, ANXA1), TCF4 downstream genes (COL1A1, COL3A1), and the mesenchymal marker Vimentin, along with a downregulation of the epithelial marker E‐Cadherin (Figure 2H,I; Figure S4M,N, Supporting Information). Additionally, Phalloidin staining of the aforementioned OSCC cells revealed a rearrangement of F‐Actin in the cytoskeleton, aligning microfilaments. Concurrently, cell morphology transitioned from a cobblestone shape to a shuttle shape, consistent with the morphological changes observed in the EMT process (Figure S4O, Supporting Information). These findings further affirmed that FOSL1 and TCF4 play pivotal roles in the “ISC‐pEMT‐CAF”‐like malignant progression of OSCC. In summary, we identified the malignant transformation pseudotime trajectory of OSF‐derived OSCC from ISC to pEMT and ultimately acquired a CAF‐like phenotype. Meanwhile, we revealed that cancer‐associated fibroblast cells (CAF2) in the tumor microenvironment were able to exacerbate the activation of the immune response, indicating their involvement in the remodeling and perpetuating of the tumor immune microenvironment.

### 2.3 ST Atlas Unveils Immune Cell Distribution and Cell–Cell Interaction Patterns in OSCC

**Figure 3:**
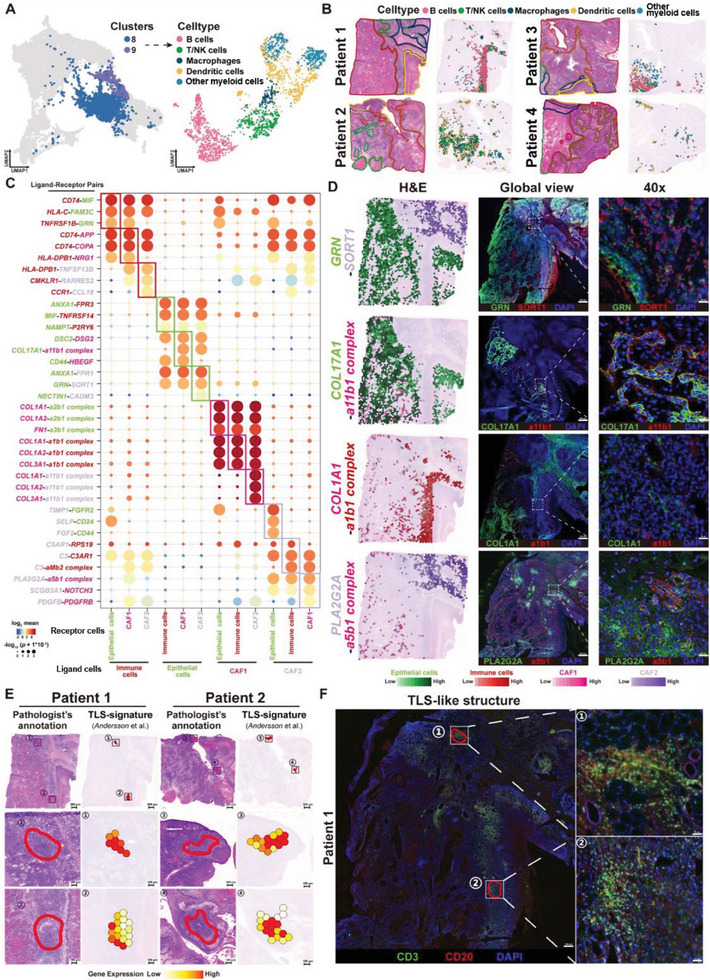
Potential Interaction among Immune Cells, Epithelial Cells and CAFs in the OSF‐derived OSCC. A) Spots corresponding to immune cells in the ST data (left, lymphocytes, and myeloid cells of C8 and C9 in the UMAP plot, with a total of 1911 spots) were extracted for a secondary reduction clustering analysis (right). Immune cells were further divided into five types, including B cells, T/NK cells, macrophages, dendritic cells, and other myeloid cells. B) ST plots displaying the spatial distribution of five types of immune cells in the tissue sections. C) Dot plots showing the three ligand–receptor pairs that are significantly expressed in each type of immune cell. The color of genes represented the cell type that genes specifically expressed in green: epithelial cells; pink: CAF1 cells; violet: CAF2 cells; and red: immune cells. D) ST feature plots exhibiting the expression level and spatial distribution of representative ligand–receptor pairs among immune cells, epithelial cells, CAF1, and CAF2 in P1. E) 2 TLS‐like regions were found in the tissue sections of P1 and P2, respectively. Left: histological features of H&E stained‐tissue sections observed under the optical microscope. Right: scores of TLS‐signature in the TLS‐like regions. Top: the whole field detected by ST; middle and bottom: local zoom of two representative TLS‐like regions. F) Multiple immunofluorescence demonstrating the spatial expression of T‐cell markers (CD3) and B‐cell markers (CD20) within the TLS‐like tissue structure in P1 (left: 10× magnification, right: 400× magnification). Green: CD3, red: CD20, blue: DAPI for nucleus.

Both OSF and OSCC are characterized by extensive immune cell infiltration.^[^ ^54^ ^]^ However, the specific roles of different immune cells in the progression of OSF‐derived OSCC still remain elusive. Therefore, we first characterized the ST features of various types of immune cells in OSF‐derived OSCC tissues. We extracted a total of 1911 spots of lymphocytes (C8) and myeloid cells (C9) from the immune subtype (Figure 1B) and categorized them into B cells, T/NK cells, macrophages, dendritic cells, and other myeloid cells (those unable to be classified into any specific myeloid cell type) by unsupervised clustering analysis (**Figure** **3**A). We analyzed the expression levels of ten characteristic genes within these five cell classes (Figure S5A, Supporting Information) and examined the expression patterns of selected genes across different immune cell subclusters (Figure S5B, Supporting Information). Our findings revealed that B cells (characteristic genes: *MZB1*, *PIM2*, *CD38*, *CD79A*) and T/NK cells (*CD3D*, *CD4*, *CD8A*, *NKG7*) exhibited relatively unique expression profiles. In contrast, dendritic cells (*CD14*, *ITGAX*, *CXCL10*, *ITGAV*), macrophages (*CD68*, *CD163*, *CXCL9*, *FCGR3A*), and other myeloid cells (*LY6D*, *S100A7*, *GPX2*, *FABP5*) shared more similar transcriptional profiles (Figure S5A,B, Supporting Information).

Next, we analyzed the distribution patterns of the Immune subtype on tissue sections and observed their significant aggregation within the Stroma region, as well as the Tumor region (Figure 3B). This suggested the presence of interactions among immune cells, tumor cells, and fibroblasts. The various ligand–receptor interactions on the cell surface play a crucial role in cell–cell communication and interactions. Using the ligand–receptor analysis algorithm *CellPhoneDB*,^[^ ^55^ ^]^ we performed ligand–receptor interaction analysis among four types of cells: epithelial cells, immune cells, CAF1 cells, and CAF2 cells. We identified a total of 739 significantly interacting ligand–receptor pairs (means > 1, *P* value < 0.001). Figure 3C displays the most significant 36 ligand–receptor pairs among these four cell types. We screened 12 representative ligand–receptor pairs within each cell and validated four of them with significant expression through immunofluorescence, namely GRN/SORT1, COL17A1/α11*β*1, COL1A1/α1*β*1, and PLA2G2A/α5*β*1 complexes (Figure 3D; Figure S5C, Supporting Information). The findings revealed significant distribution of GRN in the Tumor region. SORT1 was also expressed in the Tumor region, along with its noteworthy presence within the Stroma region, suggesting that GRN/SORT1 may facilitate cellular interactions between epithelial cells and mesenchymal‐derived fibroblasts. COL17A1 exhibited specific infiltration in the epithelial basement membranes and the leading edge of tumor–mesenchymal junctions. COL17A1 was expressed in close proximity to the integrin receptor α11*β*1 complexes within an expression neighborhood, indicating that malignant epithelial cells at the leading edge may interact with CAF1‐type cells via the COL17A1/α11*β*1 complex ligand–receptor pairs, thereby promoting the ISC‐pEMT‐CAF‐like malignant phenotype. COL1A1 demonstrated significant expression in the Stroma region and adjacency to the integrin receptor α1*β*1 complex, suggesting that CAF1‐type cells interact with immune cells through the COL1A1/α1*β*1 complex ligand–receptor pair. PLA2G2A exhibited significant expression in mesenchymal tissues and was similarly positioned next to the integrin receptor α5*β*1 complex.

We showed the expression patterns of eight other representative ligand–receptor pairs in ST as well (Figure S5D, Supporting Information). For example, the receptor–ligand pair CD74 and MIF (macrophage migration inhibitory factor), which may be involved in the interaction between immune cells and epithelial cells, has been reported to play a role in inducing tumor EMT and weakening antitumor immune activity.^[^ ^56^, ^57^ ^]^ This suggested that immune cells may induce pEMT and acquire CAF‐like phenotypes in OSF‐derived OSCC through the CD74‐mediated recognition and binding of MIF on the surface of malignant epithelial cells. Furthermore, malignant epithelial cells can suppress the antitumor immune response of immune cells through immune suppressive ligand–receptor pairs, such as *ANXA1*/*FPR3* and *NAMPT*/*P2RY6*.^[^ ^58^, ^59^, ^60^ ^]^

There are significant differences in the cell communication patterns among immune cells and different types of fibroblasts. In OSF‐derived OSCC, immune cells can express membrane MHC class II molecule HLA‐DPB1 and bind to neuregulin‐1 (NRG1) molecules on the surface of CAF1 cells, potentially influencing the EMT and fibrosis processes.^[^ ^61^, ^62^ ^]^ Besides, CAF1‐like tumor cells located at the leading edge can adhere immune cells through ligand–receptor pairs such as *COL1A1/a1b1*, *COL1A2/a1b1*, and *COL3A1/a1b1 complex*, thereby limiting the migration of immune cells into the tumor tissue.^[^ ^63^, ^64^ ^]^ Moreover, the ligand–receptor interactions between CAF1‐like tumor cells and epithelial cells (e.g., *COL1A1*/*a2b1*, *COL1A2*/*a2b1 complex*, and *FN1*/*a3b1 complex*) strengthen the immune barriers represented by collagen fibril within the Tumor region, blocking immune cell infiltration and assisting in immune evasion of OSF‐derived OSCC.

Unlike CAF1 cells, immune‐related CAF2 cells showed a spatial distribution pattern adjacent to immune cells, and they exhibit significant recruitment and activation functions toward immune cells. For example, the significant expression of the ligand *RARRES* on CAF2 enhances the expression of endogenous leukocyte chemotactic factor Chemerin, which recruits immune cells and enhances antitumor immune responses by binding to chemokine‐like receptor 1 (*CMKLR1*).^[^ ^65^, ^66^ ^]^ The significantly expressed *TNFSF13B gene* (encoding B cell activation factor *BAFF*) on CAF2 cells interacts with the MHC class II molecule *HLA‐DPB1* on the membrane of immune cells, activating the downstream biofunctions of B cells.^[^ ^67^, ^68^ ^]^ CAF2 cells recruit and activate various immune cells through other ligand–receptor pairs, such as *C5AR1/RPS19*, *CCL18/CCR1*, *C3/C3AR1*, and *C3/aMb2 complex*.^[^ ^69^, ^70^ ^]^ We performed a clinical correlation analysis of 12 representative ligand–receptor pairs in the OSCC data from TCGA and found that ligand–receptor pairs represented by GRN‐SORT1, COL17A1‐a11b1 complex, COL1A1‐a1b1 complex, and PLA2G2A‐a5b1 complex were expressed at elevated levels in OSCC compared with normal tissues and that highly expressed ligand–receptor pairs were correlated with poor prognosis in OSCC patients (Figure S5E, Supporting Information). Based on ST data, it was discovered that the complex interactions between epithelial cells, CAF1‐like tumor cells, CAF2 cells, and immune cells through ligand–receptor pairs constitute the unique immune microenvironment of OSF‐derived OSCC.

We further analyzed the patterns of ligand–receptor interaction among different types of immune cells in the immune microenvironment of OSF‐derived OSCC. We identified a total of 4,259 significantly interacting ligand–receptor pairs among macrophages, dendritic cells, T/NK cells, CAF1/2 cells, and other cells within the tumor microenvironment (Table S4 and Figure S5F, Supporting Information). Additionally, there were multiple significant ligand–receptor pairs between B cells and other immune cells, such as T/NK cells, dendritic cells, and macrophages. Considering the concentrated infiltration of various immune cells in the Stroma region of OSF‐derived OSCC, we speculated the presence of tertiary lymphoid structures (TLSs) in the Stroma region. TLSs are ectopic lymphatic structures infiltrated by germinal center B cells (GC B cells), accompanied by follicular T helper cells and follicular dendritic cells (FDCs). A high level of TLS infiltration in tumor tissues often indicates stronger antitumor immune responses and better clinical outcomes.^[^ ^71^, ^72^, ^73^ ^]^ Based on annotations by experienced pathologists, we identified four regions with TLS characteristics in sections from P1 and P2. Furthermore, in accordance with the previously published gene set of TLSs identified by ST, we found significant expression of genes associated with TLSs (TLS‐signature) in all four TLSs‐like regions (Figure 3E). These regions showed significant expression of marker genes for GC B cells (*MS4A1*, *CD38*), CD4^+^ helper T cells (*CD3D*, *CD4*), CD8^+^ effector T cells (*CD3D*, *CD8A*), FDCs (*ITGAX*, *BCL6*), and macrophages (*CD68*, *CD163*) (Figure S5G, Supporting Information). We further verified the presence of TLSs using multiple immunofluorescence staining in P1, finding that the expression characteristics of T cells (CD3) and B cell markers (CD20) were observed in two TLS‐like structures (i.e., TLS‐1 and TLS‐2) (Figure 3F), which is in line with the expression characteristics of TLS. Finally, we obtained the top 50 DEGs that significantly expressed in the TLSs‐like regions as TLS‐signature of OSF‐derived OSCC (seven DEGs were consistent with the published TLS‐signature) (log2 FC > 1, *p‐*value < 0.001) based on our ST data (Table S5, Supporting Information). By assessing the expression score of TLS‐signature of OSF‐derived OSCC in the OSCC samples (*n* = 394) from The Cancer Genome Atlas (TCGA), a high score of TLS‐signature (TLS‐high, indicating the presence of TLSs in OSCC tissues) was correlation with better overall survival of OSCC patients (Figure S5H, Supporting Information), which is consistent with previous reports in HNSCC.^[^ ^54^ ^]^ In summary, our data demonstrated the spatial characteristics of immune cell distribution and infiltration in OSF‐derived OSCC, and confirmed the intercellular interactions among epithelial cells, fibroblasts, and immune cells during the ISC‐pEMT‐CAF1 malignant progression. TLSs had been identified in OSF‐derived OSCC, and the potential value of TLSs in predicting prognosis was validated.

### 2.4 SM Atlas Reveals Metabolic Reprogramming in OSF‐Derived OSCC

**Figure 4:**
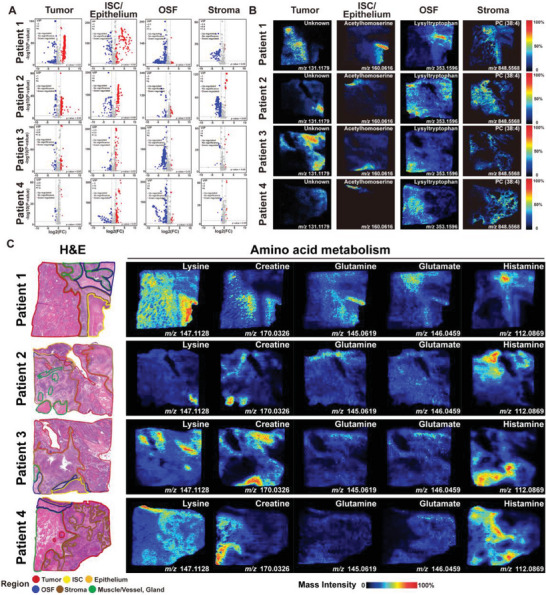
Spatial Metabolomic Atlas of OSF‐derived OSCC. A) The spatial metabolomic atlas of OSF‐derived OSCC was established by the AFADESI‐MSI platform. Representative metabolites were obtained by comparing each histopathological region. Volcano plots displayed the differential *m/z* metabolites in each histopathological region of four samples. *p*‐value < 0.05 was considered statistically significant. B) MSI images showing the abundance and distribution of representative metabolites and *m/z* information (bottom) in each histopathological region of four samples. *m/z* information was obtained in accordance with the mass spectrometry data, while several *m/z* hadn't matched specific metabolites due to the limit of metabolic databases. C) MSI images exhibiting the abundance of representative differential metabolites of amino acid metabolism in different regions of four samples.

We established the SM landscape of OSF‐derived OSCC using the AFADESI‐MSI platform. Four OSF‐derived OSCC samples were divided into 105,435 pixels (resolution: 20 × 20 µm), from which 14,673 *m/z* (mass‐to‐charge ratio) information was captured (Full MS, 70–1,000 Da) (Figure S6A, Table S6, Supporting Information). To explore the representative metabolites within different tissue regions, we annotated the 14,673 *m/z* information with metabolite annotation (detailed in the Experimental Section). By employing orthogonal partial least squares‐discriminant analysis (OPLS‐DA), we identified differentially enriched metabolites (DEMs) within each tissue region, with higher metabolite levels observed in the malignant epithelial region compared to other regions (**Figure** **4**A), consistent with metabolic reprogramming and vigorous metabolism in tumor cells during the malignant process. Subsequently, based on the histopathological annotation of each sample, we selected the ten most significantly different *m/z* information within each region (Figure S6B, Supporting Information), confirming the remarkable metabolic differences among tissue regions (refer to representative metabolites in Figure 4B). We performed metabolic pathway enrichment analysis on the DEMs and highlighted them on the metabolic pathway map (Figure S7, Supporting Information). The malignant epithelial region exhibited active amino acid metabolism, with multiple amino acid metabolic pathways converging on polyamine metabolism, primarily involving arginine and proline. Additionally, OSF and Stroma regions showed enrichment of several metabolites in pathways such as galactose metabolism, lipid metabolism (e.g., biosynthesis of unsaturated fatty acids, linoleic acid metabolism), and ABC transporters (Figure S7, Supporting Information).

Warburg effect is one of the predominant forms of energy acquisition by tumor cells, and it involves metabolic competition for carbohydrates among cells in the tumor microenvironment.^[^ ^74^, ^75^ ^]^ In OSF‐derived OSCC, the content of glucose (*m/z* 215.0317) in the malignant epithelial region was significantly lower than that in the OSF and Stroma regions. However, the metabolic products of glucose, such as oxidized gluconic acid or galactonic acid (*m/z* 195.0511), as well as intermediate metabolites of the citric acid cycle like malic acid (*m/z* 133.0143), were significantly accumulated in the malignant epithelial region (Figure S8, Supporting Information). This was consistent with the characteristic feature of tumor cells extensively consuming glucose and undergoing rapid metabolism.^[^ ^76^ ^]^ Additionally, these metabolites also contributed to the subacidity of the tumor microenvironment. Other differential metabolites in carbohydrate metabolism, such as galactose, sorbose, fructose, mannose, tagatose, myo‐inositol, and allose, had the same *m/z* ratio (*m/z* 215.0317), and could not be distinguished by AFADESI‐MSI, which is a limitation of untargeted mass spectrometry in current spatial metabolomics studies (Figure S7, Supporting Information).

Lipid metabolism is another important metabolic pathway for tumor cells to meet their high energy demands, including fatty acid metabolism, cholesterol metabolism, and phospholipid metabolism. Fatty acid *β*‐oxidation is a crucial reaction for the decomposition and energy supply of various fatty acids in tumor cells.^[^ ^77^, ^78^ ^]^ In OSF‐derived OSCC, we observed higher levels of the monounsaturated fatty acid palmitoleic acid (*m/z* 253.2174) and the essential fatty acid linoleic acid (*m/z* 279.2230) in the malignant epithelial region, suggesting that OSF‐derived OSCC cells were able to obtain energy through fatty acid *β*‐oxidation to meet their high metabolic demands during malignant progression. Acyl‐CoA metabolites, represented by acetyl‐CoA, are important products of fatty acid *β*‐oxidation in mitochondria and are involved in various metabolic pathways as well, including cholesterol biosynthesis.^[^ ^79^, ^80^ ^]^ We found that the classical metabolite of squamous cell carcinoma, cholesterol sulfate (*m/z* 465.3044), exhibited a specific accumulation and distribution in the malignant epithelial region of OSF‐derived OSCC, which may be associated with tumor cell differentiation and the inhibition of immune cell infiltration and activation.^[^ ^81^, ^82^, ^83^ ^]^ Phospholipids are important components of the cellular membrane system, and phospholipid metabolism in tumor cells has attracted attention as a potential target for cancer therapy.^[^ ^84^ ^]^ Our results revealed that phosphatidyl ethanolamine (PE (28:1), *m/z* 634.4442) had a relatively higher abundance in the Adjacent Epithelium and ISC regions, while phosphatidyl choline (PC (38:7), *m/z* 804.5539) and phosphatidyl serine (PS (38:4), *m/z* 810.5292) were remarkably enriched in the Stroma and OSF regions (Figure S8, Supporting Information). This suggested that OSF‐derived OSCC underwent phospholipid metabolic reprogramming during malignant progression to meet the increased demand for phospholipid biosynthesis for the cellular membrane system during mitosis.

Amino acid metabolism serves a dual role in both metabolic activity and protein biosynthesis, and tumor cells have a much higher demand and consumption of amino acids compared to normal epithelial cells.^[^ ^85^, ^86^ ^]^ In OSF‐derived OSCC, amino acid metabolites such as aspartate (*m/z* 133.0303), citrulline (*m/z* 198.0850), arginine (*m/z* 175.1189), spermidine (*m/z* 146.1652), and spermine (*m/z* 203.2230) accumulated clearly in the malignant epithelial region, indicating active polyamine metabolism and metabolic reprogramming characteristics in OSF‐derived OSCC (Figure 4C).

### 2.5 Active Polyamine Metabolism is a Metabolic Hallmark in OSF‐Derived OSCC

**Figure 5:**
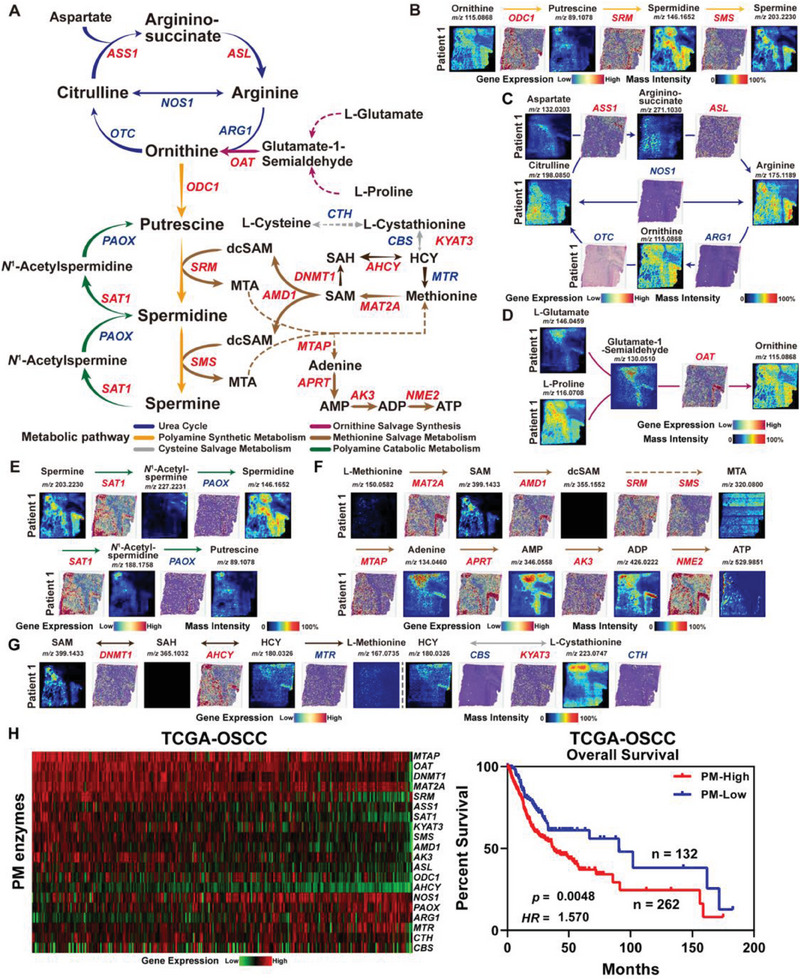
Metabolic Reprogramming of Polyamine Metabolism in OSF‐derived OSCC. A) Schematic maps of polyamine metabolism, including urea cycle (blue arrows), polyamine synthetic metabolism (yellow arrows), ornithine salvage synthesis (purple arrows), polyamine catabolic metabolism (green arrows), methionine salvage metabolism (light and dark brown arrows), and cysteine salvage synthesis (grey arrows). Essential metabolites and enzymes (italic) involved in the whole polyamine metabolism in OSF‐derived OSCC were marked in the maps. Red: high expression in malignant epithelial regions; blue: low expression in malignant epithelial regions. B) The spatial abundance or expression of essential metabolites and related enzymes in the polyamine synthetic metabolism (yellow arrows in Figure 5A) of P1. C) The spatial distribution feature of metabolic products and enzymes in the urea cycle (blue arrows in Figure 5A) of P1. D) The ST and SM feature of ornithine salvage synthesis (purple arrow in Figure 5A) in P1. E) The spatial multi‐omic feature of polyamine catabolic metabolism (green arrows in Figure 5A) in P1. F) The spatial multi‐omic characteristic of methionine salvage metabolism (SAM‐dcSAM pathway; light brown arrows in Figure 5A) in P1. G) The spatial multi‐omic characteristic of methionine salvage synthesis (SAM‐SAH‐HCY pathway; dark brown arrows in Figure 5A) and cysteine salvage synthesis (grey arrows in Figure 5A) in P1. H) Expression and overall survival of PM enzymes in the TCGA‐OSCC cohort (*n* = 394). Left: expression level of PM enzymes from high (red) to low (green); right: Kaplan‐Meier curve of OSCC patients with different expression levels of PM enzymes.

Polyamine metabolism is one of the important nitrogen‐containing metabolic pathways in the human body. Aberrant polyamine metabolism not only affects the regulation of metabolic flux in tumor cells but also extensively influences various malignant biological processes, such as cell survival and proliferation, through its involvement in intracellular epigenetic modifications.^[^ ^87^, ^88^ ^]^ The schematic diagram of the amino acid metabolism pathway with polyamine metabolism as its core is depicted in **Figure** **5**A. By integrating ST and SM data, we found that in the polyamine metabolic flux of OSF‐derived OSCC, the metabolic products putrescine, spermidine, and spermine, as well as the related enzymes ornithine decarboxylase 1 (ODC1), spermidine synthase (SRM), and spermine synthase (SMS), exhibited specific distribution in the Tumor region. This suggests that abnormal polyamine metabolism is a prominent metabolic phenotype in OSF‐derived OSCC and may play a driving role in the malignant progression of the oral epithelium (Figure 5B; Figure S9A, Supporting Information).

We further analyzed the synthesis and degradation of polyamines in OSCC based on the metabolic pathway (Figure 5A). The urea cycle is an upstream synthetic metabolic pathway for polyamines.^[^ ^89^, ^90^ ^]^ The ST results showed low expression of metabolic enzymes involved in the direct conversion between arginine, ornithine, and citrulline (i.e., *ARG1/2*, *OTC*, *NOS1*). Only the enzyme argininosuccinatelyase (ASL) in the urea cycle, which is involved in the regulation of aspartate, and its metabolite argininosuccinate showed similar spatial distribution patterns (Figure 5C; Figure S9B, Supporting Information). This suggests that the overall metabolism of the urea cycle is relatively low in OSF‐derived OSCC, and ornithine may be maintained in tumor cells through other compensatory pathways. For example, previous reports suggested that glutamate‐1‐semialdehyde derived from glutamate and proline metabolism serves as a supplementary precursor metabolite of ornithine.^[^ ^91^ ^]^ In OSF‐derived OSCC, the precursor metabolite, glutamate‐1‐semialdehyde, and the corresponding enzyme, ornithine aminotransferase (OAT), showed spatial distribution patterns similar to that of ornithine, suggesting that the synthesis of ornithine in OSF‐derived OSCC mainly relied on the glutamate–proline metabolism pathway (Figure 5D; Figure S9C, Supporting Information). Under normal physiological conditions, intracellular polyamines are primarily excreted in the acetylated form, and a portion of acetylated polyamines can be oxidized to spermine or spermidine to maintain intracellular polyamine levels.^[^ ^92^ ^]^ The region‐specific expression of spermidine/spermine N1‐acetyltransferase 1 (SAT1) in OSF‐derived OSCC promoted the acetylation of polyamines in tumor cells (Figure 5E; Figure S9D, Supporting Information). Acetylspermine and acetylspermidine showed significant differences in distribution and abundance in the OSF‐derived OSCC, and the expression level of polyamine oxidase (PAOX) was low, suggesting that acetylspermine was mainly excreted from the cell, rather than being supplemented through a PAOX‐dependent pathway for spermidine levels. As a precursor metabolite of putrescine, acetylspermidine showed similar spatial distribution and abundance to putrescine, but the expression level of PAOX might limit the synthesis of putrescine (Figure 5E; Figure S9D, Supporting Information).

We analyzed the impact of polyamine metabolic reprogramming on other amino acid metabolic pathways. S‐adenosylmethionine (SAM), an important metabolite of the methionine salvage pathway, participates in the synthesis of downstream polyamine products by providing propylamine substrates (Figure 5A).^[^ ^93^, ^94^ ^]^ In the malignant epithelial region of OSF‐derived OSCC, a similar spatial distribution was observed between SAM and its downstream metabolites (spermidine, spermine), and the expression of their associated enzymes (AMD1, SRM, SMS), which may be caused by the activation of the salvage pathway induced by low levels of methionine in the tissue (Figure 5F; Figure S9E, Supporting Information). As one of the metabolic products of polyamine synthesis, 5′‐methylthioadenosine (MTA) plays an important role in both the methionine salvage pathway and the ATP salvage pathway.^[^ ^95^, ^96^ ^]^ In the OSF‐derived OSCC, the hydrolysis of MTA to adenine could be catalyzed by phosphatases such as APRT, AK3, and NME2, and used for the salvage synthesis of ATP, further indicating that polyamine metabolism synergistically activated the methionine salvage pathway to meet the energy demands during the malignant progression of OSF‐derived OSCC (Figure 5F; Figure S9E, Supporting Information). Notably, intracellular levels of methionine can also be supplemented through homocysteine (HCY),^[^ ^97^, ^98^ ^]^ and there is a reversible metabolic balance between SAM, S‐adenosyl‐L‐homocysteine (SAH), and HCY (Figure 5G; Figure S9F, Supporting Information). However, the low expression level of methionine synthase methyltetrahydrofolate–homocysteine methyltransferase (MTR) in the samples also suggested that the methionine salvage pathway in OSF‐derived OSCC was mainly complemented through the SAM‐decarboxylated SAM (dcSAM) pathway, rather than the SAM‐SAH pathway. In addition, the accumulation of HCY in the OSF‐derived OSCC was also related to the transamination of l‐cystathionine, which was limited by the expression of the metabolic enzyme cystathionine gamma‐lyase (CTH), indicating a relatively little contribution to the synthesis of L‐Cysteine (Figure 5G; Figure S9F, Supporting Information). Based on a microarray data of betel nut‐associated OSCC set (GSE37991), we performed clinical analysis of polyamine metabolic pathway‐related enzymes to further confirm that ten metabolic enzymes were significantly upregulated in betel nut‐associated OSCC and eight metabolic enzymes were significantly downregulated in betel nut‐associated OSCC (Figure S9G, Supporting Information).

Finally, we validated the functional correlation between the expression of 23 polyamine metabolic enzymes (PM enzymes) and the prognosis of OSCC. By analyzing RNA‐seq data from OSCC patients in TCGA, we found that higher expression of polyamine metabolic enzymes was associated with poorer prognosis in patients (Figure 5H), consistent with previous reports on the promotion of tumor development by polyamine metabolism.^[^ ^87^, ^88^ ^]^ In summary, our results collectively suggested that active polyamine metabolism was an important metabolic feature of OSF‐derived OSCC, and its metabolic reprogramming could lead to adverse prognostic events in the OSCC.

## 3 Conclusion

Betel nut is one of the most popular addictive substances, with ≈600 million people worldwide chewing various forms of betel nut products.^[^ ^99^, ^100^ ^]^ Chewing betel nut is a well‐known risk factor for OSF, which clinically presents as progressive restricted mouth opening and severely affects the quality of life. During the progression of OSF, excessively activated fibroblasts interact with immune cells and epithelial cells in a complex manner, ultimately leading to “inflammation‐cancer” malignant transformation and the development of OSCC. However, different from other premalignant lesions (i.e., oral leukoplakia), various subtypes of OSCC arising from OSF have not yet been defined, and the key molecular events during the malignant progression are still not clear. The spatial characteristics of metabolic reprogramming within the tumor microenvironment of OSF‐derived OSCC have not been reported.

Through ST analysis, our effort is the first to clarify the molecular subpopulations and the heterogeneity of tumor cells in the OSF‐derived OSCC. We aim to explore the process and patterns of OSCC development by conducting pseudo‐time analysis on these cells. The Cancer Genome Atlas Network has previously proposed the criteria of molecular subpopulations for HNSCC based on traditional bulk RNA‐seq data. It classifies HNSCC into classical, atypical, mesenchymal, and basal subtypes, which exhibit obvious differences in oxidative stress, cell death, and innate immunity response.^[^ ^101^ ^]^ However, due to the limitations of bulk RNA‐seq technology in dissecting the tumor microenvironment, it can only achieve subpopulation classification of biopsy tissue samples from different HNSCC patients. It cannot reveal the heterogeneity of tumor cells within the same tissue or their evolutionary patterns at single‐cell resolution. Besides, due to a lack of spatial information in their scRNA‐seq data, the previous study did not further investigate the origins, evolutionary relationships, biological characteristics, and spatial interactions of different subpopulations of tumor cells, CAF1 and CAF2 cells. Based on the aforementioned cellular subpopulation criteria, we propose a novel malignant transformation process model for OSF‐derived OSCC called “ISC‐pEMT‐CAF like phenotype”, which shows that in the early stages of tumorigenesis, tumor cells exhibit active proliferation and differentiation capabilities, accompanied by stress and hypoxia features. While developing into invasive carcinoma, HNSCC cells begin to show functional differences in proliferation, translation, and immune response. Interestingly, late‐stage OSF‐derived OSCC demonstrates significant extracellular matrix remodeling ability, characterized by elevated expression of EMT‐associated genes and increased collagen deposition, resembling fibroblast‐like functions. This suggests that OSF‐derived OSCC may induce tumor cells to acquire fibroblast‐like functions by exerting tumor phenotypic plasticity.^[^ ^102^ ^]^

Recently, Sun et al.^[^ ^103^ ^]^ elucidated the evolution of cellular composition within the tumor microenvironment during the progression from oral leukoplakia (OLK) to early‐stage OSCC (T1 stage), employing scRNA‐seq and ST sequencing technologies. OLK is another precancerous lesion, typically unrelated to betel nut chewing. Sun et al. gathered a cohort of nine patients with OLK‐associated OSCC, encompassing 5 for scRNA‐seq, 3 for ST sequencing, and one case analyzed using both single‐cell RNA‐seq and ST sequencing. None of the nine samples had a history of betel quid chewing, with two cases having a concurrent history of alcoholism and smoking. All nine patients were classified as early stage (T1N0M0). In our current study, we incorporated four patients with OSF‐derived OSCC, characterized by a prolonged history of betel quid chewing and a clinical diagnosis of OSF. Notably, all tumor tissues were categorized as TNM stage T3, indicating clinically advanced OSCC. We, along with Sun et al., conducted spatial omics analysis on OSCC stemming from different etiologies. Sun et al. observed a gradual enhancement of EMT and other pathways during the initiation of oral leukoplakia‐associated OSCC, progressing from normal epithelial tissue, through atypical hyperplastic epithelial tissue, to tumor tissue. This observation aligns with the “ISC‐pEMT‐CAF” malignant phenotype identified in our study. Both studies revealed a significant increase in cell interaction between epithelial cells and surrounding mesenchymal cells from the stage of atypical hyperplastic epithelial tissue to the tumor tissue. This suggests that the augmentation of EMT and cell interaction is a shared characteristic in the malignant progression of OSCC, irrespective of the causative factors.

Fibroblasts are a core component of the tumor microenvironment, and their interactions with tumor cells and immune cells can affect various malignant phenotypes such as tumor proliferation, invasion, and immune evasion.^[^ ^104^ ^]^ pEMT cells exhibit active expression of both epithelial and mesenchymal markers and are located at the leading edge of the tumor and adjacent stroma, often considered the “pioneers” of tumor invasion and metastasis.^[^ ^105^ ^]^ In OSF‐derived OSCC, we discovered that CAF1 represents a group of cells that have undergone full EMT and acquired a CAF‐like phenotype. They are mainly distributed at the leading edge between the Tumor and Stroma regions and exhibit more active extracellular matrix remodeling capability. Meanwhile, CAF2 represents myofibroblast‐like cells recruited and induced by tumor cells. They are primarily located in the stroma and OSF regions and exhibit immune activity such as complement activation and innate immune response. Their functions align more with the inflammatory cancer‐associated fibroblasts, particularly the apCAFs characteristics.^[^ ^106^, ^107^, ^108^ ^]^ pEMT, CAF1, and CAF2 cells undergo a transition from epithelial keratinization to extracellular matrix remodeling and immune response regulation through the expression of various TFs such as *FOSL1*, *TCF4*, and *SPI1*. The interaction between tumor cells and CAF1 cells involves ligand–receptor pairs such as the *COL17A1/a11b1* complex, which collectively promote the conversion of pEMT tumor cells to CAF1‐like cells. Additionally, CAF1 cells interact with various immune cells through ligand–receptor pairs such as *COL1A1/a1b1*, leading to adhesion and restriction of immune cell infiltration, thereby assisting in immune evasion in OSF‐derived OSCC. However, despite the presence of significantly expressed ligand–receptor pairs in tumor cells, CAF1, and CAF2 cells (e.g., *GRN/SORT1*, *PLA2G2A/a5b1* complex), there is a lack of spatial proximity between CAF1 and CAF2 cells. Conversely, immune cells and CAF2 cells possess the feasibility of both spatial distance and expression of ligand–receptor pairs. We speculate that CAF2 cells are more likely to interact with immune cells in the Stroma region through ligand–receptor pairs such as *C5AR1/RPS19*, *C3/C3AR1*, and *C3/aMb2 complex*, thereby synergistically constructing the unique immune microenvironment features of OSF‐derived OSCC.

Sun et al.^[^ ^103^ ^]^ identified distinct subpopulations of CAF cells, monocytes, and Treg cells and delineated their functional characteristics in the initiation of oral leukoplakia‐associated OSCC. Utilizing the fibroblast subpopulation classifications proposed by Li et al.^[^ ^109^ ^]^ and Mao et al.,^[^ ^110^ ^]^ Sun et al. identified Mesen_CAF (similar to myofibroblasts with representative genes such as *POSTN*, *ASPN*, *TNC*, and *WNT5A*), Infla_CAF (similar to inflammatory fibroblasts with representative genes including hormone and metabolism‐related genes like *CXCL12*, *IGF1*, and *CFD*), and Cycling_CAF (similar to proliferative fibroblasts with representative genes including FAP, S100A4, and VIM). The focus was primarily on summarizing the functional characteristics of the predominant Mesen_CAF subpopulation cells, including TGF*β*, EMT, angiogenesis, and the PI3K‐AKT‐mTOR signaling pathway, as well as cellular communication involving ligand–receptor pairs such as CSF1‐CSF1R, TNC‐a4b1 complex, and TGF*β*‐TGF*β*R. In contrast, our study identified two fibroblast subtypes, namely the CAF1 subtype (represented by genes including *POSTN*, *FN1*, *COL1A1*) and the CAF2 subtype (represented by genes including *CXCL12*, *C3*, *PLA2G2A*), based on the fibroblast subtype identification in head and neck squamous carcinoma by Puram et al.^[^ ^24^ ^]^ We characterized the CAF1 subtype (represented by genes including *POSTN*, *FN1*, and *COL1A1*) and described their spatial distribution characteristics. Notably, our findings indicated that CAF1 subtype cells primarily exhibited extracellular matrix functions such as extracellular matrix remodeling, collagen deposition, and cell adhesion, while CAF2 subtype cells were predominantly associated with immune regulatory functions like complement activation, antimicrobial humoral immunity, and immune response. In terms of cellular communication, CAF1 subtype cells engaged with immune cells and epithelial cells through interactions like COL1A1‐a1b1 complex and FN1‐a3b1 complex. On the other hand, CAF2 subtype cells interacted with immune cells and epithelial cells through C5AR1‐RPS19 and SELP‐CD24, contrasting with the results of cellular communication analysis by Sun et al. Given that collagen deposition is a prominent clinical pathological feature of OSF, and fibroblast overactivation significantly influences collagen production and metabolism, the observed differences in CAF phenotypes between OSF‐derived and OLK‐associated OSCC tissues warrant further exploration. A comprehensive investigation into the microenvironmental distinctions between these two types of OSCC tissues, considering the impact of CAF itself, intercellular communication, and the effects of CAF on collagen metabolism, would be valuable.

The prognosis of OSCC patients varies significantly depending on the etiology of the disease, with generally better outcomes observed in OSF‐derived OSCC.^[^ ^111^, ^112^ ^]^ We hypothesize that this is related to the immune cell status and immune response levels in the tumor tissues. In OSF‐derived OSCC, we found that various types of immune cells exhibit characteristics of local infiltration in the adjacent OSF and Stroma region of the tumor. These immune cells often form TLSs with B cells or T/NK cells as the core, consistent with the histological feature of TLS. TLSs are ectopic lymphoid structures formed after chronic inflammatory stimulation, primarily consisting of B cell and T cell zones, and infiltrated by myeloid cells such as dendritic cells and macrophages. The high infiltration level of TLSs in tumor tissues often indicates a stronger antitumor immune response.^[^ ^72^, ^113^, ^114^ ^]^ Furthermore, immunofluorescence experiments confirmed the presence of TLS in the Stroma region of OSF‐derived OSCC. Additionally, using the TLS signature, a set of genes associated with TLS, we scored OSCC patients from the TCGA dataset. Patients with high expression of TLS‐signature genes had better prognoses, consistent with previous reports in other HNSCC. For instance, the favorable prognosis observed in HPV‐positive HNSCC may be associated with active immune infiltration and TLS infiltration.^[^ ^54^, ^115^, ^116^ ^]^ Similarly, OSCC patients with an OSF background also exhibit better outcomes,^[^ ^117^, ^118^, ^119^ ^]^ although there are currently no reports on its immune landscape and specific mechanisms contributing to improved prognosis. We speculate that as a “hot” tumor arising from chronic uncontrollable inflammation, OSF‐derived OSCC is characterized by an inflammatory response that contributes to abundant immune infiltration in the Stroma region. The interaction between CAF2 and various types of immune cells through ligand–receptor pairs may be a key factor in maintaining the immune response level in OSF‐derived OSCC. Additionally, cell communications among immune cells, tumor cells, and CAF1 play a crucial role in tumor immune evasion. In summary, exploring the immune cell infiltration patterns in OSF‐derived OSCC provides valuable guidance for future research on the mechanisms of immune evasion during the malignant transformation process from OSF to OSCC.

Metabolic reprogramming stands out as a fundamental hallmark in the malignant progression of tumors.^[^ ^120^, ^121^ ^]^ In the context of malignant progression in OSCC derived from OSF, specific expression patterns were observed in key enzyme molecules related to lipid metabolism (PLA2G2E, APOE), amino acid metabolism (ODC1), and glucose metabolism (LDHA). To delve deeper into the metabolic reprogramming features within the microenvironment of OSF‐derived OSCC and their correlation with tumor malignant progression, we utilized AFADESI‐MSI technology to obtain spatial metabolomic profiles for the first time. Our findings revealed a significantly lower glucose content in Tumor regions compared to paracancerous stroma and OSF tissues, with glucose metabolites exhibiting significant aggregation. This confirms that OSF‐derived OSCC extensively utilizes and consumes glucose. The elevated glucose content in CAF, serving as the “soil” for tumor growth, suggests a potential co‐regulation of glucose metabolism in the tumor microenvironment by CAF and tumor cells, thereby facilitating tumor development.^[^ ^122^, ^123^, ^124^ ^]^ Lipid metabolism represents another critical pathway for tumor cells to acquire energy.^[^ ^125^ ^]^ In OSF‐derived OSCC, our observations indicate a preference for tumor cells to derive energy through the fatty acid *β*‐oxidation pathway, aligning with findings reported in other tumors.^[^ ^126^, ^127^ ^]^

Amino acid metabolism is an important nitrogen metabolic pathway in the tumor microenvironment, and tumors often undergo amino acid metabolic reprogramming to meet the increased nitrogen metabolic demands.^[^ ^128^ ^]^ Since Leeuwenhoek^[^ ^129^ ^]^ first discovered spermine in 1678, polyamine metabolism has been recognized as a crucial part of intracellular nitrogen metabolism. The elevated levels of intracellular polyamine pool play a significant role in the malignant proliferation and growth of tumor cells.^[^ ^130^, ^131^ ^]^ The intracellular synthesis of polyamines begins with ornithine, an amino acid that does not participate in protein formation. In OSF‐derived OSCC, we found that the urea cycle, which is involved in polyamine synthesis, is significantly suppressed, and the tumor microenvironment relies more on the glutamine–aspartate pathway to supplement ornithine. Previous studies have also reported the inhibition of the urea cycle, which is required for ornithine synthesis, in various epithelial‐derived tumors.^[^ ^132^, ^133^, ^134^, ^135^ ^]^ Owing to their unique polycationic structure, polyamines can exert epigenetic regulatory functions through various mechanisms, including electrostatic binding, induction of post‐translational hypusination modification, and activation of specific signaling pathways.^[^ ^136^, ^137^, ^138^ ^]^ Increasing evidence suggests that polyamines can also regulate the fate of immune cells in the tumor microenvironment.^[^ ^88^, ^139^ ^]^ In OSF‐derived OSCC, we observe a negative correlation between polyamine levels and immune cell infiltration, providing spatial evidence for the important role of polyamines in immunosuppressive microenvironments. Although there are many mature and safe inhibitors of polyamine synthesis enzymes available currently (e.g., ODC inhibitor DFMO) and even with a Phase I clinical trial (NCT03536728) of polyamine blockade therapy ongoing (e.g., AMXT 1501+DFMO), it should be acknowledged that the complexity of the immune microenvironment remains a major limiting factor for the efficacy of polyamine metabolism blockade methods.^[^ ^87^ ^]^ Recently, more research has focused on achieving metabolic reprogramming of “cold” tumors by blocking polyamine metabolism, thereby effectively enhancing the immune response in the tumor microenvironment.^[^ ^140^, ^141^, ^142^ ^]^ Considering the metabolic regulatory role of the oral microbiota, further investigation is warranted to understand the specific mechanisms of microbiota‐mediated polyamine metabolism in the development of epithelial‐derived tumors and immune evasion.

In addition, in the OSF‐derived OSCC tumor microenvironment, part of the ligand–receptor pairs not only play a communication function in inducing various types of cellular interactions but also function in synergistically activating a variety of metabolic processes. For example, CD74 may affect the level of glucose metabolism within the tumor tissue area by binding to the receptor MIF molecule on epithelial cells, possibly by activating downstream glucose catabolism.^[^ ^143^ ^]^ GRN on the surface of tumor cells can activate the glycolytic process in Treg cells by interacting with TNFRSF1B receptors on the surface of immune cells (Treg cells), resulting in increased glucose depletion and elevated accumulation of lactic acid in the mesenchymal area infiltrated by immune cells.^[^ ^144^ ^]^ The immune cell membrane molecule CMKLR1, after interacting with RARRES2, a receptor on the surface of CAF2 cells, activates lipid metabolism in CAF2 cells, resulting in the accumulation of large amounts of lipid‐associated metabolites in the mesenchymal area of fibroblast aggregates and the OSF area.^[^ ^145^ ^]^

In conclusion, we pioneered the application of spatial multi‐omics techniques to reveal the ST and SM characteristics of OSF‐derived OSCC and dissected the transcriptomic and metabolic profiles of tumor cells, CAF1/2, and immune cells. We proposed the malignant progression process of “ISC‐pEMT‐CAF‐like phenotype” in OSF‐derived OSCC and explored the infiltration characteristics of immune cells during this process, as well as the ligand–receptor communication patterns between immune cells and the tumor microenvironment. Furthermore, we unveiled, for the first time, metabolic reprogramming in the malignant progression of OSF‐derived OSCC, with a hallmark shift in polyamine metabolism. Through the integration of ST and SM data, we discovered the aberrant expression of polyamine metabolism enzymes driving the polyamine metabolic reprogramming in OSF‐derived OSCC, providing direct evidence for the role of polyamine metabolism in promoting immune evasion and sustaining tumor development. Based on the spatial multi‐omics exploration of polyamine metabolism, our work shed new light on further understanding the mechanisms of immune evasion in OSF‐derived OSCC and provided new clues for targeted clinical therapies.

## 4 Experimental Section

### OSF‐Derived OSCC Clinical Samples

Four OSF‐derived OSCC samples were collected during surgical resection at the Second Xiangya Hospital of Central South University (Changsha, Hunan, China). This study was approved by the Joint Ethics Committee of the Second Xiangya Hospital of Central South University (No. 2023‐Z0196). Written consent was obtained from each patient before the start of this study. All oral mucosa in OSF‐derived OSCC had white streak‐like mottled manifestations and restricted mouth opening. Tissue sections with Masson's staining confirmed that all four patients were OSF‐derived OSCC patients. Diagnoses of all specimens were confirmed by two senior pathologists according to the histopathological examination. TNM and clinical stages were defined in accordance with the eighth edition of the American Joint Committee on Cancer staging system. Specifically, each sample contained tumor tissues, adjacent nonmalignant tissues, and distal nonmalignant tissues. Fresh OSCC samples were embedded in pre‐chilled optimal cutting temperature (OCT) compound after drying and snap frozen in liquid nitrogen and stored at −80 °C for ST and AFADESI‐MSI analysis. Detailed information for these OSF‐derived OSCC clinical samples is listed in Table S1 (Supporting Information).

### Cell Culture, Plasmids, and Transfection

The OSCC cell lines CAL27 and HN30 used in this study were purchased from the ATCC cell bank. The cell lines were cultured in vitro using DMEM medium supplemented with 10% fetal bovine serum and 1% penicillin–streptomycin dual antibody and placed in a cell culture incubator containing 5% CO_2_ under 37 °C.

pcDNA3.1‐FOSL1‐3× Flag (NM_005438), pcDNA3.1‐TCF4‐MYC (NM_001083962), and the control empty plasmid were purchased from YouBio (YouBio Co, China). Plasmids were transfected into CAL27 and HN30 cell lines using Neofect DNA transfection reagent (NEOFECT biotech, China).

### RNA Extraction and Quantitative Real‐Time PCR (qRT‐PCR)

Total RNA was extracted from cells using Trizol reagent (AG Co, China). cDNA was obtained by reverse transcription of RNA using HiScript II RT Reverse Transcription Kit (Vazyme, China) following the instructions. qRT‐PCR was performed according to the manual of SYBR Green qPCR MasterMix Kit (Selleck, U.S.A.) on a CFX real‐time PCR detection system equipped with CFX Manager TM software (Version 3.1, Bio‐Rad). *β*‐Actin was used as an endogenous control and the relative expression of target RNAs was calculated using the 2‐∆∆CT method. The primers used are listed in Table S7 (Supporting Information).

### Western Blotting

Cells were lysed using RIPA buffer (Beyotime, China) containing 25× protease inhibitor mixture (Keygen, China) to extract total cellular proteins. The concentration of each sample was determined using a BCA assay kit (Bio‐Rad, U.S.A.). Protein samples were separated using an 8–10% SDS‐PAGE gel and then transferred onto a PVDF membrane (Millipore, U.S.A.). The PVDF membranes were blocked with 5% milk for 1 h at room temperature and incubated overnight at 4 °C with primary antibodies. Subsequently, the PVDF membranes, after washing with PBS, were incubated with the secondary antibody at 37 °C for 1 h. Target protein expression was visualized using the NcmECL Ultra kit (NCM, China). *β*‐Actin was used as an endogenous reference. The antibodies used are listed in Table S8 (Supporting Information).

### 10× Genomics Visium ST Sequencing—Slide Preparation

OCT‐embedded OSCC samples, prechilled in the cryostat microtome for 30 min, were cut and placed in the 10× Visium spatial slides printed with four identical capture areas (6.5 × 6.5 mm). Each spot in the slide had a diameter of ≈55 µm with six surrounding spots nearby.

### 10× Genomics Visium ST Sequencing—Fixation, H&E Staining, and Imaging

Fixation, H&E staining, and imaging were performed as previously described (10× Genomics, Protocol #CG000160). In short, sectioned slides were incubated in precooled (at −20 °C) methanol (Millipore Sigma, Darmstadt, Germany) and isopropanol (Millipore Sigma). Then, slides, at room temperature, were incubated in Mayer's hematoxylin (Dako, Agilent, Santa Clara, CA) for 7 min, stained in Bluing loading buffer (Dako) for 2 min, and Eosin (Millipore Sigma) diluted 1:10 in Tris‐base (ThermoFisher Scientific, 0.45 m, pH 6.0) for 1 min. Slides were washed in Milli‐Q water (Corning, Corning, NY) after each of the staining steps. Each capture area on the slides was imaged in a bright field. Raw images were processed via 3DHISTECH (3DHISTECH Ltd., Budapest, Hungary).

### 10× Genomics Visium ST Sequencing—Tissue Permeabilization

After fixation and staining, the tissue slides were placed in the slide cassettes, adding 70 µL permeabilizing enzymes (10× Genomics) in each well (except wells of the positive and negative controls). The slides were incubated at 37 °C for different times (3, 6, 12, 18, 24, and 30 min). Each well of samples was washed in 100 µL 0.1× SSC (Sigma–Aldrich, St. Louis, MO) after removing permeabilizing enzymes. After tissue permeabilization, released mRNA was captured by specific probes on the slides and reversely transcribed into fluorescently labeled cDNA. The optimal permeabilization time for this procedure was determined by the intensity of the fluorescent signal.

### 10× Genomics Visium ST Sequencing—Reverse Transcription, Spatial Library Construction, and Sequencing

Tissue sections were permeabilized using permeabilizing enzymes at optimal permeabilization times to release mRNA from cells in the tissue sections. Reverse transcription was performed on PCR instrument (MyCycler, Bio‐Rad, Hercules, CA) following the 10× Genomics protocol (Protocol # CG000238). After reverse transcription, cDNAs in the supernatant layer were collected for synthesis, inactivation, in vitro transcription, and adaptor ligation, the products from which were used in a second reverse transcription for the construction of the spatial library. Sequencing of the spatial library was performed on the Illumina NovaSeq 6000 platform with an average of 4329 spots per sample and 76 211 reads per spot.

### 10× Genomics Visium ST Sequencing—Data Processing

The 10× Visium ST raw data were processed with the Space Ranger pipeline (10× Genomics). Specifically, considering the ST parameter including total spots, total reads per spot, counts, and UMIs, feature‐barcode matrices were generated by aligning reads to the human genome GRCh38 with Space Range (Version 1.0.0). Then the data was scaled and normalized via the function scTransform to analyze the high variance characteristics.^[^ ^142^ ^]^ R package *Harmony* was used to correct the batch effect of expression profiles.^[^ ^146^ ^]^ PCA) was performed to linearly reduce the dimension of the matrix, while the FindNeighbors, FindClusters, and RunUMAP *Seurat* functions performed unsupervised clusters and visualized the PCA‐reduced data. Based on the 11 clusters derived from unsupervised clustering analysis, the R package *Seurat* (Version 4.0) was used to perform differential expression analysis to find out the DEGs of each cluster. DEGs were then filtered with log_2_ FC > 0.25 and *p‐value* < 0.05. The spatial expression pattern of DEGs was visualized with the SpatialPlot *Seurat* function.

### Cell Type Identification

A public OSCC scRNA‐seq (GSE195832) as reference data set into our ST profile via *SPOTlight* ^[^ ^35^ ^]^ to determine the proportions of cell types in each spot was mapped. Then, the NormalizeData and ScaleData *Seurat* functions were used to preprocess the scRNA‐seq data. Subsequently, RunPCA, FindNeighbors, FindClusters, and RunUMAP functions were used for dimensionality reduction, clustering, and visualization. Cell types were annotated by the *singleR*. Marker genes of each cell type were derived by the FindAllMarkers *Seurat* functions for the cluster–markers matrix. ST data was deconvoluted by the spotlight_deconvolution *SPOTlight* function based on non‐negative matrix factorization (NMF). Eventually, the proportion of cell types in each spot was visualized by the spatial_scatterpie function. The final cell type in each spot was determined in accordance with the first proportion principle.

### Pseudotime Analysis

Pseudotime analysis was conducted by the *Monocle2* ^[^ ^146^ ^]^ to simulate the dynamic characteristics of cell types, region types, and gene expression during tumorigenesis of OSF‐derived OSCC. Initially, malignant epithelial cells were extracted and normalized by the s.CellDataSet, estimateSizeFactors, estimateDispersions of *Monocle2* functions for the construction of tumorigenesis pseudotime tree (TPT). Next, DEGs of TPT were obtained by the differentialGeneTest, setOrderingFilter functions to build a minimum spanning tree (MST). The malignant pseudo time trajectory of OSF‐derived OSCC was obtained based on the integration of TPT and MST. Based on the characteristics of malignant trajectory, branched expression analysis modeling (BEAM) was established to analyze the transcriptomic patterns and cell fates of each branch via the plot_genes_branched_pseudotime function.

### Cell Enrichment Score

Cell enrichment score was assessed in accordance with the gene sets of tumor cell subpopulations in the public data set. In brief, the *Seurat* function AddModuleScore was conducted to score the top 100 signatures in cell types of OSF‐derived OSCC.^[^ ^147^ ^]^ The subpopulation scores were log2 normalized for the visualization of subpopulation scores in each sample. The correlation among each cell subpopulation was calculated by the rcorr function of R package *Hmisc*.

### Transcription Factor Analysis

The transcription factors (TFs) and their regulation in pEMT, CAF1, and CAF2 were analyzed by *pySCENIC* (Version 0.12.0) (https://github.com/aertslab/SCENIC).^[^ ^148^ ^]^ In detail, GRNBoost2 was used to obtain the regulons (TFs and their target genes) co‐expression modules. The cisTarget was performed to identify the interaction between TFs and targeted genes. AUCell was used to quantify the activity of regulons. Finally, the results were visualized in R with *ggplot2* package.

### Ligand–Receptor Interaction Analysis

Ligand–receptor interaction network among epithelial cells, fibroblasts, and immune cells was inferred by C*ellPhoneDB* (Version 2.1.7) as described before.^[^ ^55^ ^]^ Initially, the expression and interaction of ligand–receptor pairs were obtained via the cellphonedb method statistical_analysis function in the conda environment of Python (Version 3.9). The differentially expressed ligand–receptor pairs were filtered with a cutoff of average expression log2 mean > 1 and *p‐value* < 0.001. The cell interaction network was imaged by the *igraph* and *qgraph* R package with the number of ligand–receptor pairs noted. The top three differentially expressed ligand–receptor pairs were used for the dot plot visualized by the *ggplot2* R package. Finally, the SpatialDimPlot *Seurat* function was employed to present the spatial expression of ligand–receptor pairs.

### The Identification and Functional Analysis of TLS‐Like Regions

First, two senior pathologists verified that only P1 and P2 have tertiary lymphoid structure (TLS)‐like regions. In addition, the transcriptomic characteristics of TLS‐like regions were confirmed by the TLS signature introduced by Andersson et al.^[^ ^149^, ^150^ ^]^ Next, the SpatialFeaturePlot function was performed to reveal the expression of marker genes in TLS‐like regions. Finally, *limma* and *Seurat* R package (FindMarkers function) were used for the analysis of the top 50 TLS‐signature with a cutoff of log2 FC > 1 and adjusted *p‐value* < 0.001, which are listed in Table S5 (Supporting Information) in detail. Prognostic analysis was conducted based on the TCGA‐OSCC cohort (*n* = 394) with all normal samples excluded. The TCGA‐OSCC cohort was divided into two groups: TLS‐High (above the cutoff value) and TLS‐low (below the cutoff value). GraphPad Prism (Version 8.0.0) was used to generate the Kaplan–Meier plots of TCGA‐OSCC for the comparison of the overall survival. Log Rank (Mantel‐Cox) was performed to calculate the *p‐value* (<0.05) and hazard ratio (HR).

### Cell Immunofluorescence

Cells were cultured on slides in 12‐well culture plates at a density of ≈10–15% and then fixed with 4% paraformaldehyde for 10 min at room temperature. Next, cells were permeabilized with Saponin and blocked with 5% BSA for 30 min at room temperature. Slides were incubated with primary antibody overnight at 4 °C. Secondary antibody was added to the washed slides and incubated for 30 min at room temperature. After staining with 4′,6‐diamidino‐2‐phenylindole (DAPI), the anti‐quenching sealer was added dropwise and sealed with a coverslip. Cells were imaged using a confocal laser scanning microscope (STELLARIS 5, Leica, USA).

### Multiplex Immunofluorescence of Tissue Samples

Multiple immunofluorescence staining of OSF‐derived OSCC tissue sections was performed using the Triple Label Multiplex Immunofluorescence Kit (AWI0693, Abiowell, China). Briefly, frozen sections were fixed and then treated with Saponin to break the membrane. The sections were incubated with 10× EDTA antigen repair buffer (SL1860, Coolaber, China) at 60 °C for 30 min. Endogenous peroxidase blocker and BSA blocking solution were added sequentially for 25 min each, and primary antibodies (ligand molecule or CD3 primary antibody) were added dropwise to the tissue sections and incubated at 4 °C overnight. On the following day, the secondary antibody was added dropwise after washing and incubated at room temperature away from light. Following a 5 min reaction with TSA‐520 fluorescent dye (Abiowell, China), the preceding round of unbound antibodies was washed away, and another droplet of the primary antibody (receptor molecule or CD20) was added dropwise. The secondary antibody was then incubated and reacted with TSA‐570 fluorescent dye (Abiowell, China), and the nuclei were stained with DAPI and shielded with a droplet of anti‐quenching blocker. Images were captured using a confocal laser scanning microscope (STELLARIS 5, Leica, USA).

### Airflow‐Assisted Desorption Electrospray Ionization‐Mass Spectrometry Imaging (AFADESI‐MSI)—Slide Preparation

Cryostat microtome (Leica CM 1950, Leica Microsystem, Germany) was used to section the OCT‐embedded OSF‐derived OSCC tissues with 10 µm thickness. The tissue sections were mounted onto the positive charge ionizing microscope slide, dehydrated at −20 °C for 1 h, and stored at temperature for 2 h before AFADESI‐MSI analysis. Additionally, a neighbor section was chosen for H&E staining to annotate the histological regions.

### Airflow‐Assisted Desorption Electrospray Ionization‐Mass Spectrometry Imaging (AFADESI‐MSI)—Detection

AFADESI‐MSI analysis was conducted as described in previously published research.^[^ ^151^ ^]^ In brief, dried sections were mounted on the AFADESI‐MSI platform (Beijing Victor Technology Co., LTD, Beijing, China), which was equipped with an AFADESI ion source and Q‐Orbitrap mass spectrometer (Q Exactive, Thermo Scientific, U.S.A.) for both positive‐ and negative‐ion mode. The tissue sections were ionized at atmospheric pressure (flow rate: 5 µL min^−1^, sprayer voltage: 7 kV) with a mixture of acetonitrile and water (8:2) as the spray solvent. The distance between the sprayer‐transport tube and the sprayer‐section surface was 3 mm. The flow rate of transporting gas was 45 L min^−1^ in the transport tube with a 3 kV transporting voltage and a 350 °C capillary temperature. The metabolites of tissue sections were scanned with the S‐lens voltage setting at 55 V, automated gain control as 2E6 mode, and the maximum injection time as 200 ms. Samples were scanned continuously in the *x* direction at a rate of 200 µm s^−1^, and a 100‐µm separated space in the *y* direction. The range of obtained *m/z* was between 70 and 1000 Dalton with a 70 000 Dalton resolution.

### Airflow‐Assisted Desorption Electrospray Ionization‐Mass Spectrometry Imaging (AFADESI‐MSI)—Data Processing

Raw data (.raw files) was converted into the imML format available for the MSiReader software (MSI Software Solutions, LLC, North Carolina, USA.), an open‐access MSI software based on the Matlab platform, to construct an overlap between MSI and histological images. The overlap images were utilized to obtain the distribution characteristics of mass spectrometry profiles. The annotation of tissue regions, including Tumor, Stroma, OSF, Adjacent Epithelium, and ISC region, were presented with H&E staining in the Supporting Information. The OPLS‐DA was performed to construct the correlation model of metabolites in different tissue regions. The contribution of metabolites to the differences among tissue regions was assessed by the variable importance of projection (VIP) value. Detailed information concerning the scanned *m/z* is shown in the Supporting Information. Two‐tail Student's *t‐test* was performed to confirm the statistical significance of DEMs among tissue regions. DEMs were set to meet the following statistical criteria: VIP > 1.0, |log2(FC) > 1| and *p‐value* < 0.05.

### Airflow‐Assisted Desorption Electrospray Ionization‐Mass Spectrometry Imaging (AFADESI‐MSI)—Metabolites Annotation

Metabolites detected by AFADESI‐MSI were annotated by comparing the extracted adducted ions with the smetDB metabolomic database (Shanghai Luming Biological Technology Co.Ltd), the public Human Metabolome Database (https://hmdb.ca/), Metlin (https://metlin.scripps.edu/), and LIPID MAPS (https://www.lipidmaps.org/). The *m/z* ratio of all metabolites annotated was less than 10 ppm (part per million) compared with their monoisotopic molecular weight (MMW).

### Statistical Analysis

R (Version 4.2.0) and GraphPad Prism (Version 8.0) were used for statistical analysis. Raw data derived from ST were log2 transformed and normalized for further analysis. Data in this study were presented as mean ± SD and the sample size for each statistical analysis was at least three. Two‐tail Student's *t*‐test (*n* ≤ 2) and one‐way ANOVA (*n* > 2) were performed to analyze the parameter data. The Vlnplot function of the *Seurat* R package was used to generate the violin plots. The Raincloud plot was generated online at BioLadder (https://www.bioladder.cn/). A *p* *‐*value of less than 0.05 was considered statistically significant.

## Conflict of Interest

The authors declare no conflict of interest.

## Author Contributions

Y.Z., Q.W., and M.Z. contributed equally to this work. Z.J.G., W.X., and Z.Y.Z. conceived ideas and designed the project. Y.Z., Q.W., and M.X.Z. conducted most data analysis with assistance from Z.J.G., W.X., and Z.Y.Z. in generating the analyzing strategy. Y.Z., Q.W., and M.X.Z. conducted cell biological and histopathological experiments with the help of Z.J.G., W.X., and Z.Y.Z. in designing experiments. Y.Z., M.X.Z., S.S.Z., K.Y.L., and L.S.L. collected clinical samples for ST and SM analysis with guidance from C.M.F., P.C., S.Q.F., Q.J.L., C.G., F.Y.W., Z.J.G., W.X., and Z.Y.Z. designed the computational analysis. J.S.G., Q.J.Y., L.S., S.Q.F., and Q.J.L. diagnosed all clinical samples according to the histopathological examination. Y.Z. wrote the original manuscript with assistance from Q.W., M.X.Z., S.S.Z., J.S.G., C.M.F., P.C., C.G., and F.Y.W. Besides, Z.J.G., W.X., and Z.Y.Z. revising and editing the manuscript. Z.J.G., W.X., and Z.Y.Z. take responsibility for research supervision and funding acquisition. All authors approved the submission of the final manuscript.

## Supporting information

Supporting Information

## Data Availability

The raw and processed expression data of ST that support the findings of this study are openly available in Gene Expression Omnibus at https://www.ncbi.nlm.nih.gov/geo/query/acc.cgi?acc=GSE220978, reference number 220978. Raw metabolomic data detected by AFADESI‐MSI is available in the OMIX, China National Center for Bioinformation/Beijing Institute of Genomics, Chinese Academy of Sciences (https://ngdc.cncb.ac.cn/omix/) with accession number OMIX003566.
