# Supplementary material for: Spatial Transcriptomic and Metabolomic Landscapes of Oral Submucous Fibrosis‐Derived Oral Squamous Cell Carcinoma and its Tumor Microenvironment
Source: Adv Sci (Weinh). 2024 Jan 16;11(12):2306515. doi: 10.1002/advs.202306515 (PMC10966560; doi:10.1002/advs.202306515)
Supplement: Supplementary file 1 — Supporting Information [file ADVS-11-2306515-s001.pdf]

ADVANCED SCIENCE
Open Access

Supporting Information

for *Adv. Sci.*, DOI 10.1002/advs.202306515

Spatial Transcriptomic and Metabolomic Landscapes of Oral Submucous Fibrosis-Derived Oral Squamous Cell Carcinoma and its Tumor Microenvironment

Yuan Zhi, Qian Wang, Moxin Zi, Shanshan Zhang, Junshang Ge, Keyue Liu, Linsong Lu, Chunmei Fan, Qijia Yan, Lei Shi, Pan Chen, Songqing Fan, Qianjin Liao, Can Guo, Fuyan Wang, Zhaojian Gong, Wei Xiong* and Zhaoyang Zeng**

·Supporting Information

Spatial Transcriptomic and Metabolomic Landscapes of Oral Submucous Fibrosis-derived Oral Squamous Cell Carcinoma and its Tumor Microenvironment

Yuan Zhi[1, 2#,] Qian Wang[2, 3#], Moxin Zi[1, 2#], Shanshan Zhang[4], Junshang Ge[3], Keyue Liu[1], Linsong Lu[1], Chunmei Fan[3], Qijia Yan[4], Lei Shi[1], Pan Chen[2], Songqing Fan[1], Qianjin Liao[2], Can Guo[3], Fuyan Wang[3], Zhaojian Gong[1,3], Wei Xiong[1,2,3*], Zhaoyang Zeng[1,2,3*]*

Figure S1 | Histological Examination of OSF-derived OSCC Samples

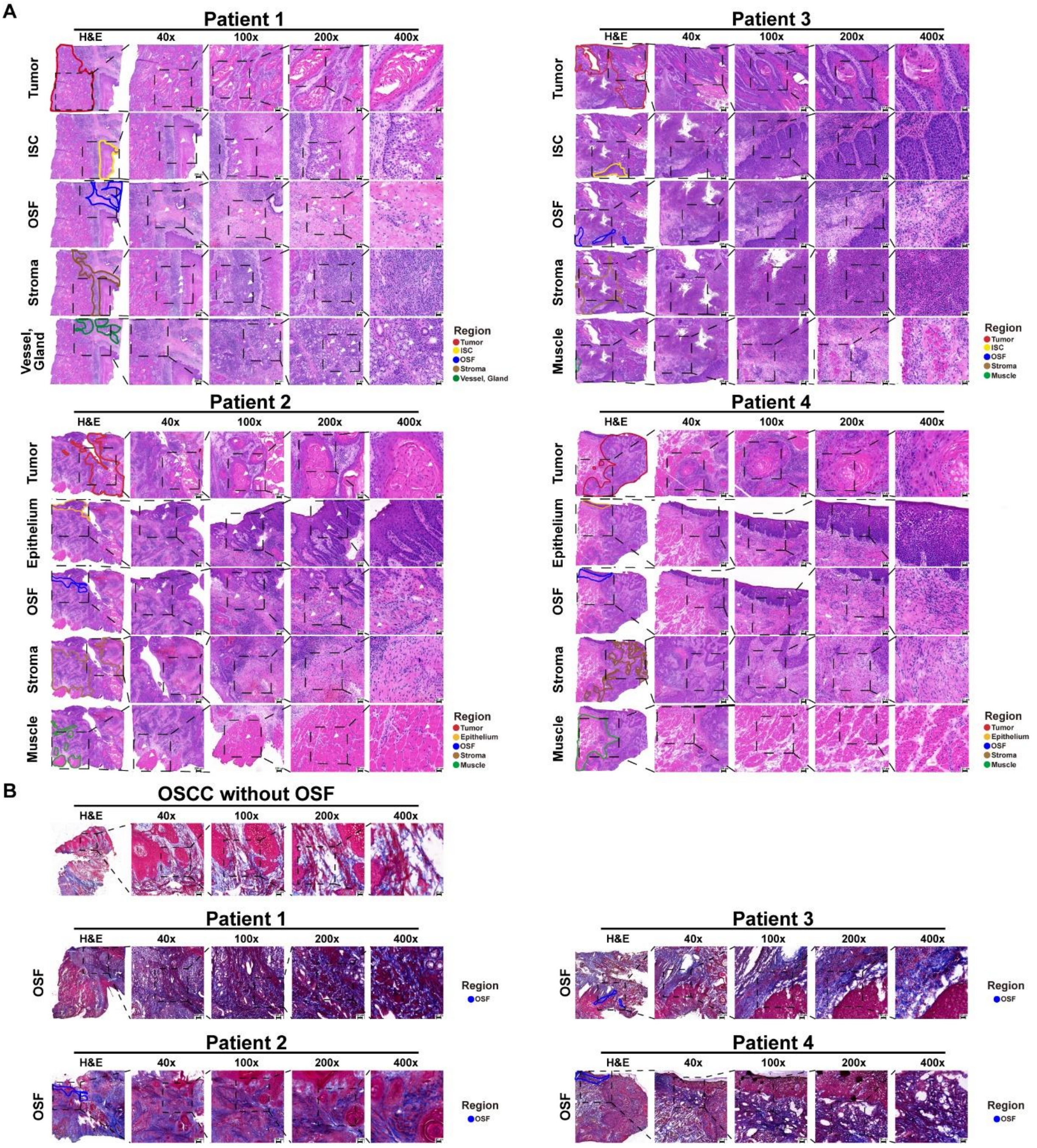

(A) After H&E staining, representative fields (6.5 mm × 6.5 mm) of 4 OSF-derived OSCC were selected for ST and SM analysis in accordance with the guidance of senior pathologists. There are Tumor

(red line surrounded), ISC (yellow line, only in P1 and P3), OSF (blue line), Stroma (brown line), Muscle (green line, P2-P4) and Vessel/Gland (green line, only in P1), Adjacent Epithelium (orange line, only in P2 and P4) regions in the 4 samples. Representative panoramic images (6.5 mm × 6.5 mm) and enlarged views of certain histopathological regions (from left to right: 40×, 100×, 200×, 400×) were presented (from left to right) with scale bars of 200 μm, 100 μm, 50 μm, and 20 μm, respectively.

(B) Masson's Staining revealed the deposition of collagen fibers in OSF-derived OSCC. 4 cases of OSF-derived OSCC showed significant blue staining of collagen fibers in the OSF region (blue line). However, no collagen fiber deposition was observed in the adjacent epithelial tissue of OSCC without OSF, which was used as a negative control. From left to right, representative panoramic images of the field of view (6.5 mm × 6.5 mm), and 40×, 100×, 200×, and 400× magnification images of the corresponding histopathological areas with scales of 200 μm, 100 μm, 50 μm, and 20 μm, respectively.

Figure S2 | ST Characteristics of OSF-derived OSCC Samples

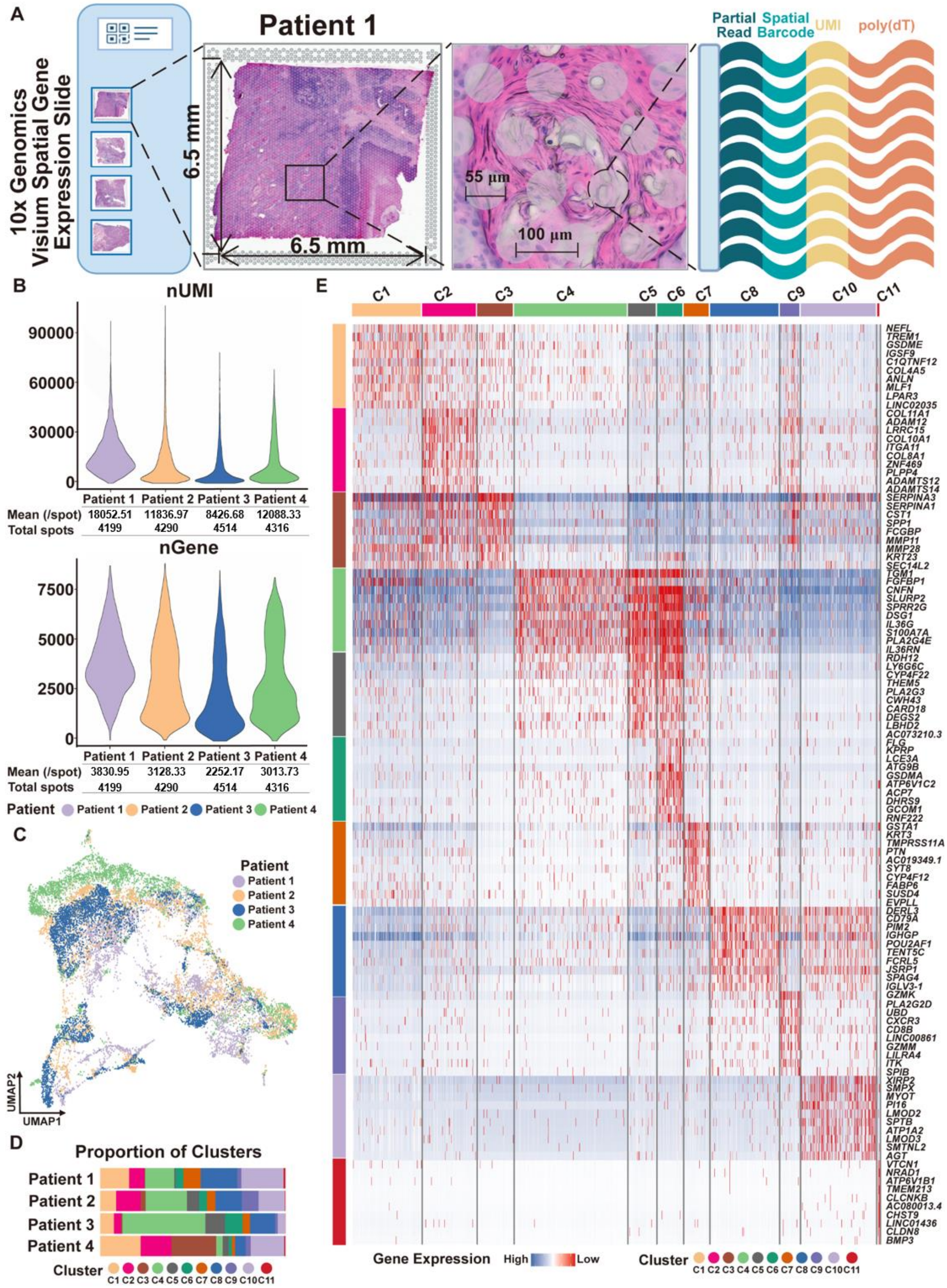
A
10x Genomics Visium Spatial Gene Expression Slide
Patient 1
6.5 mm
6.5 mm
55 µm
100 µm
Partial Read
Spatial Barcode
UMI
poly(dT)
B
nUMI
90000
60000
30000
0
Patient 1 Patient 2 Patient 3 Patient 4
Mean (/spot) 18052.51 11836.97 8426.68 12088.33
Total spots 4199 4290 4514 4316
nGene
7500
5000
2500
0
Patient 1 Patient 2 Patient 3 Patient 4
Mean (/spot) 3830.95 3128.33 2252.17 3013.73
Total spots 4199 4290 4514 4316
Patient Patient 1 Patient 2 Patient 3 Patient 4
C
Patient
Patient 1
Patient 2
Patient 3
Patient 4
UMAP2
UMAP1
D
Proportion of Clusters
Patient 1
Patient 2
Patient 3
Patient 4
Cluster C1 C2 C3 C4 C5 C6 C7 C8 C9 C10 C11
E
C1 C2 C3 C4 C5 C6 C7 C8 C9 C10 C11
NEFL
TREM1
GSDME
IGSF9
C1QTNF12
COL4A5
ANLN
MLF1
LPAR3
LINC02035
COL11A1
ADAM12
LRRC15
COL10A1
ITGA11
COL8A1
ZNF469
PLPP4
ADAMTS12
ADAMTS14
SERPINA3
SERPINA1
CST1
SPP1
FCGBP
MMP11
MMP28
KRT23
SEC14L2
TGM1
FGFBP1
CNFN
SLURP2
SPRR2G
DSG1
IL36G
S100A7A
PLA2G4E
IL36RN
RDH12
LY6G6C
CYP4F22
THEM5
PLA2G3
CWH43
CARD18
DEGS2
LBHD2
AC073210.3
FLG
KPRP
LCE3A
ATG9B
GSDMA
ATP6V1C2
ACP7
DHRS9
GCOM1
RNF222
GSTA1
KRT3
TMPRSS11A
PTN
AC019349.1
SYT8
CYP4F12
FABP6
SUSD4
EVPLL
DERL3
CD79A
PIM2
IGHGP
POU2AF1
TENT5C
FCRL5
JSRP1
SPAG4
IGLV3-1
GZMK
PLA2G2D
UBD
CXCR3
CD8B
LINC00861
GZMM
LILRA4
ITK
SPIB
XIRP2
SMPX
MYOT
PI16
LMOD2
SPTB
ATP1A2
LMOD3
SMTNL2
AGT
VTCN1
NRAD1
ATP6V1B1
TMEM213
CLCNKB
AC080013.4
CHST9
LINC01436
CLDN8
BMP3
Gene Expression High Low
Cluster C1 C2 C3 C4 C5 C6 C7 C8 C9 C10 C11

(A) Schematic diagram showing the strategy of ST. In P1, frozen tissue section (6.5 mm × 6.5 mm) was divided into 4,316 spots (about 55 μm in diameter, 100 μm between 2 neighboring spots) for ST sequencing. After permeabilization, total RNA in each spot was captured by primers with specific spatial barcode attached on the 10x Visium Spatial Gene Expression Slide for reverse transcription. Eventually, cDNA was eluted from the slides for further sequencing.

(B) Violin plots displaying the average numbers of unique molecular identifiers (nUMI, top) and gene expression (nGene, bottom) in each spot of 4 samples.

(C) 17,319 spots obtained from ST were performed unsupervised clustering analysis UMAP and the distribution of each spot in the UMAP plot was colored by patients. There was a balanced distribution among spots from 4 samples, indicating the absence of batch effect among 4 OSF-derived OSCC patients.

(D) Bar plots showing the proportion of 11 clusters (obtained from the UMAP analysis) in each patient.

(E) 10 representative marker genes of each cluster were obtained through differential expressed gene (DEG) analysis. Heatmap showed the expression patterns of the top 10 marker genes in each cluster.

Figure S3 | Cell Constitution Analysis of Each Spot in OSF-derived OSCC

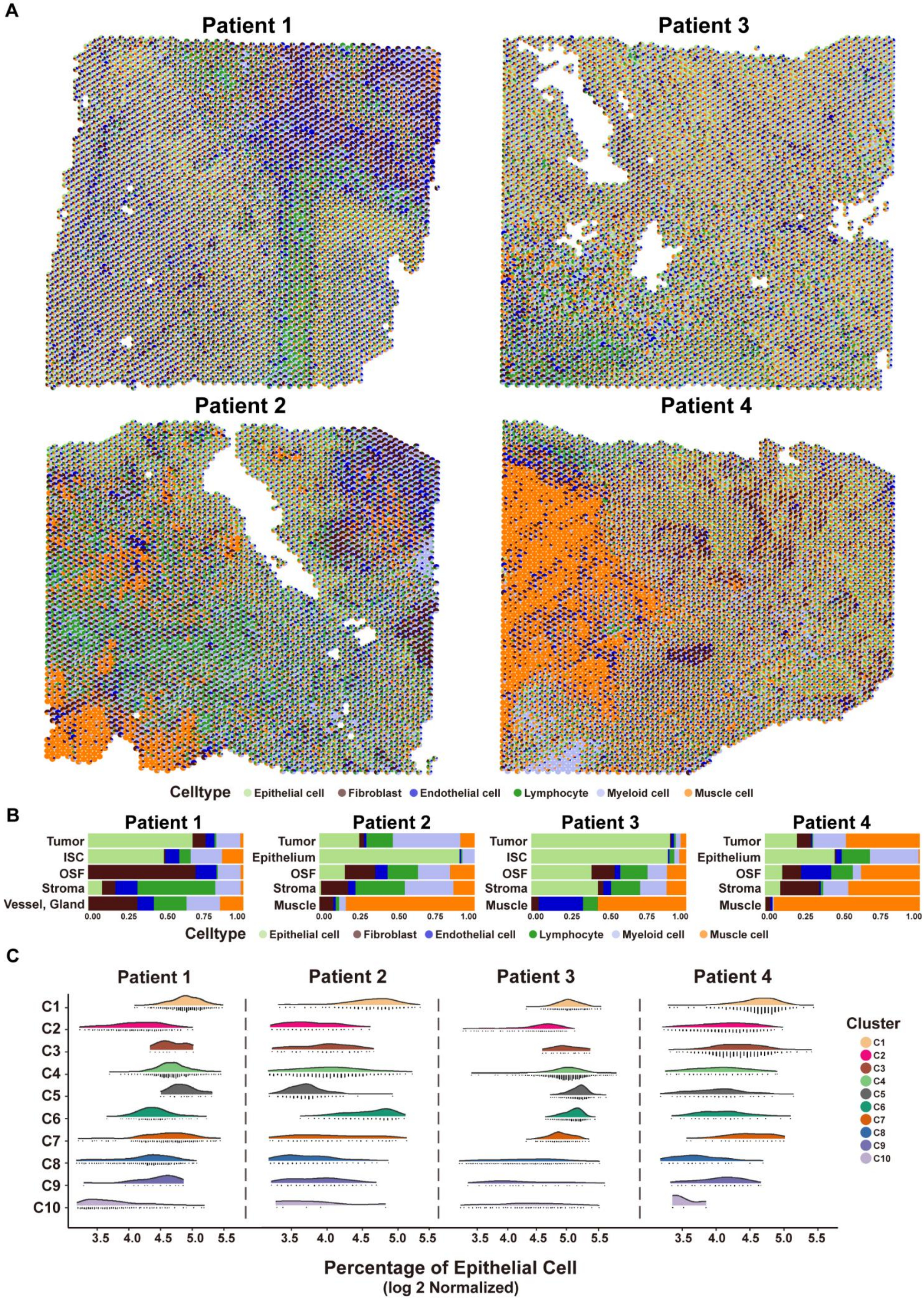

(A) Given the limited resolution (about 55 μm in diameter) and complex cell constitution (about 2-30 cells per spot) in one spot, we integrated a public OSCC scRNA-seq data (GSE195832). *SPOTlight* was performed to analyze the constitution and proportion of annotated cell types in each spot of 4 OSF-derived OSCC samples. The constitution of cells included Epithelial cells (light green), Fibroblasts (brown), Endothelial cells (blue), lymphocytes (bright green), myeloid cells (violet), and muscle cells (orange).
(B) Bar plots showing the proportion of annotated cell types in each histopathological region (annotated and presented in Fig. S1 according to the guidance of senior pathologists) of 4 samples.
(C) Raincloud plots presenting the percentage (log2 normalized) of epithelial cells in each cluster of 4 samples.

Figure S4 | Spatio-temporal Evolution and Expression Patterns of Malignant Epithelial Cells in OSF-derived OSCC

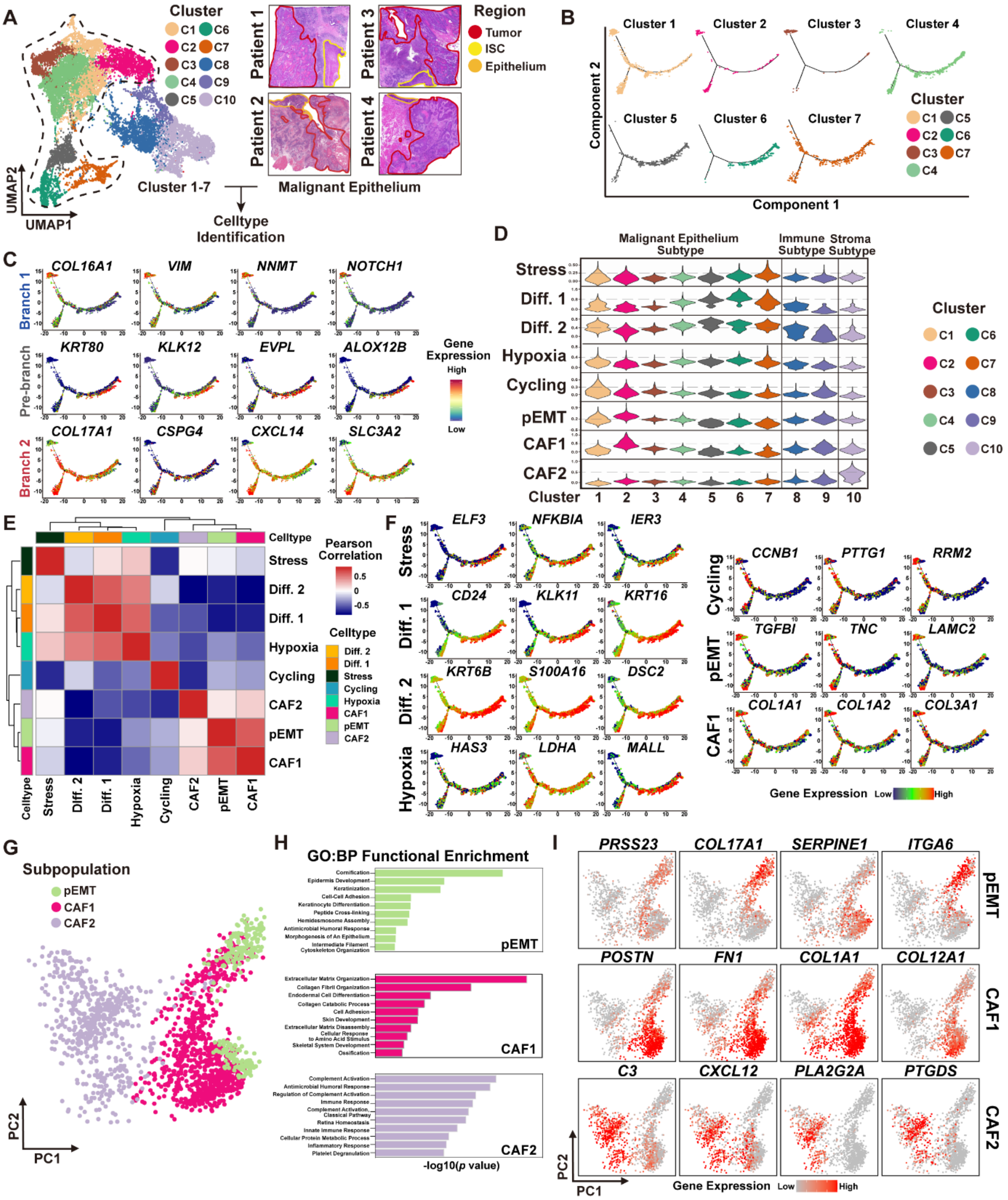
A
Cluster
Region
Tumor
ISC
Epithelium
Patient 1
Patient 2
Patient 3
Patient 4
Cluster 1-7
Malignant Epithelium
Celltype Identification
UMAP1
UMAP2
B
Component 1
Component 2
C
D
Malignant Epithelium Subtype
Immune Subtype
Stroma Subtype
Stress
Diff. 1
Diff. 2
Hypoxia
Cycling
pEMT
CAF1
CAF2
E
Pearson Correlation
Celltype
F
G
Subpopulation
H
GO:BP Functional Enrichment
-log10(p value)
I
Gene Expression

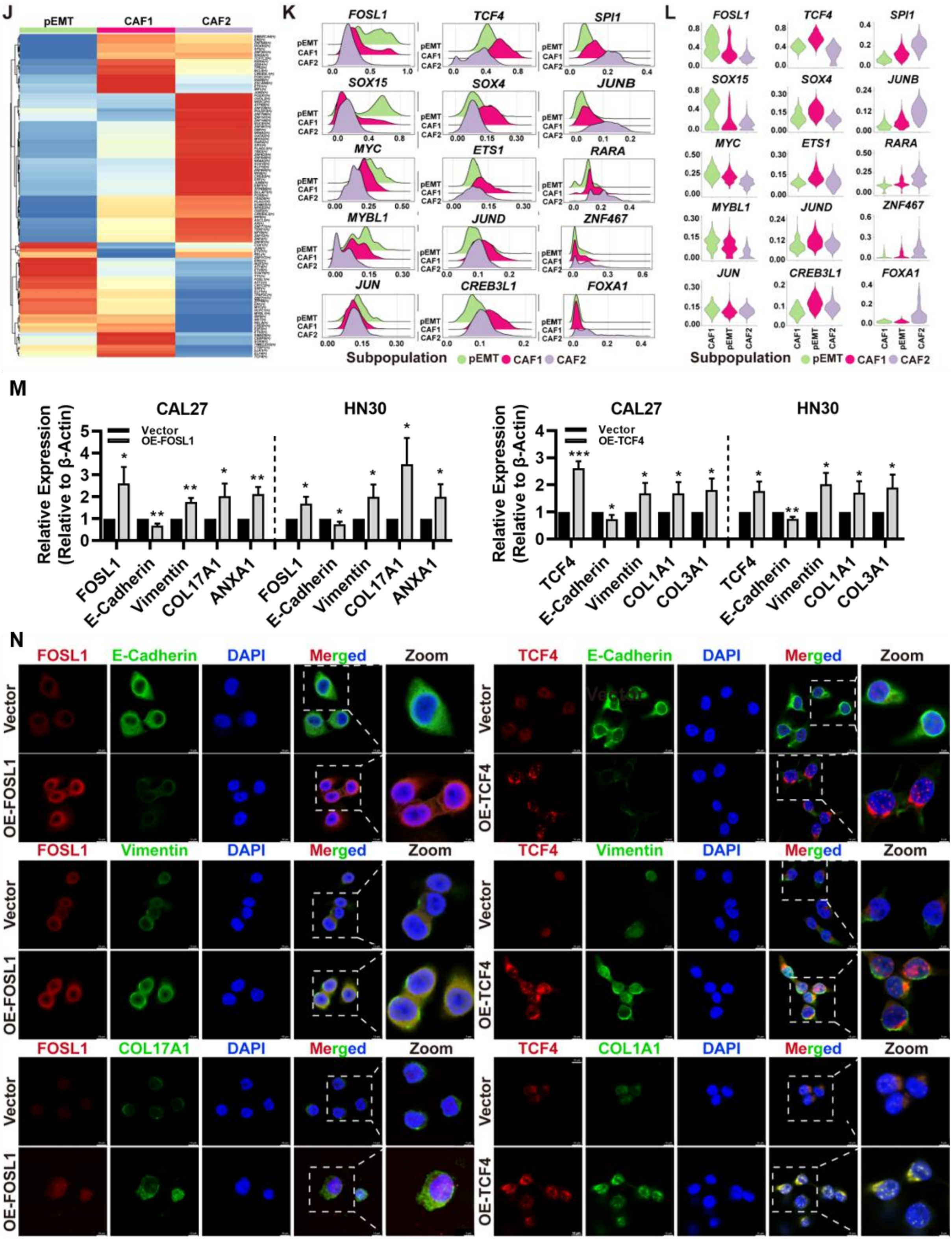
J
pEMT
CAF1
CAF2
K
FOSL1
TCF4
SPI1
SOX15
SOX4
JUNB
MYC
ETS1
RARA
MYBL1
JUND
ZNF467
JUN
CREB3L1
FOXA1
Subpopulation pEMT CAF1 CAF2
L
M
CAL27
HN30
Relative Expression (Relative to β-Actin)
Vector
OE-FOSL1
OE-TCF4
FOSL1
E-Cadherin
Vimentin
COL17A1
ANXA1
TCF4
COL1A1
COL3A1
N
DAPI
Merged
Zoom
Vector

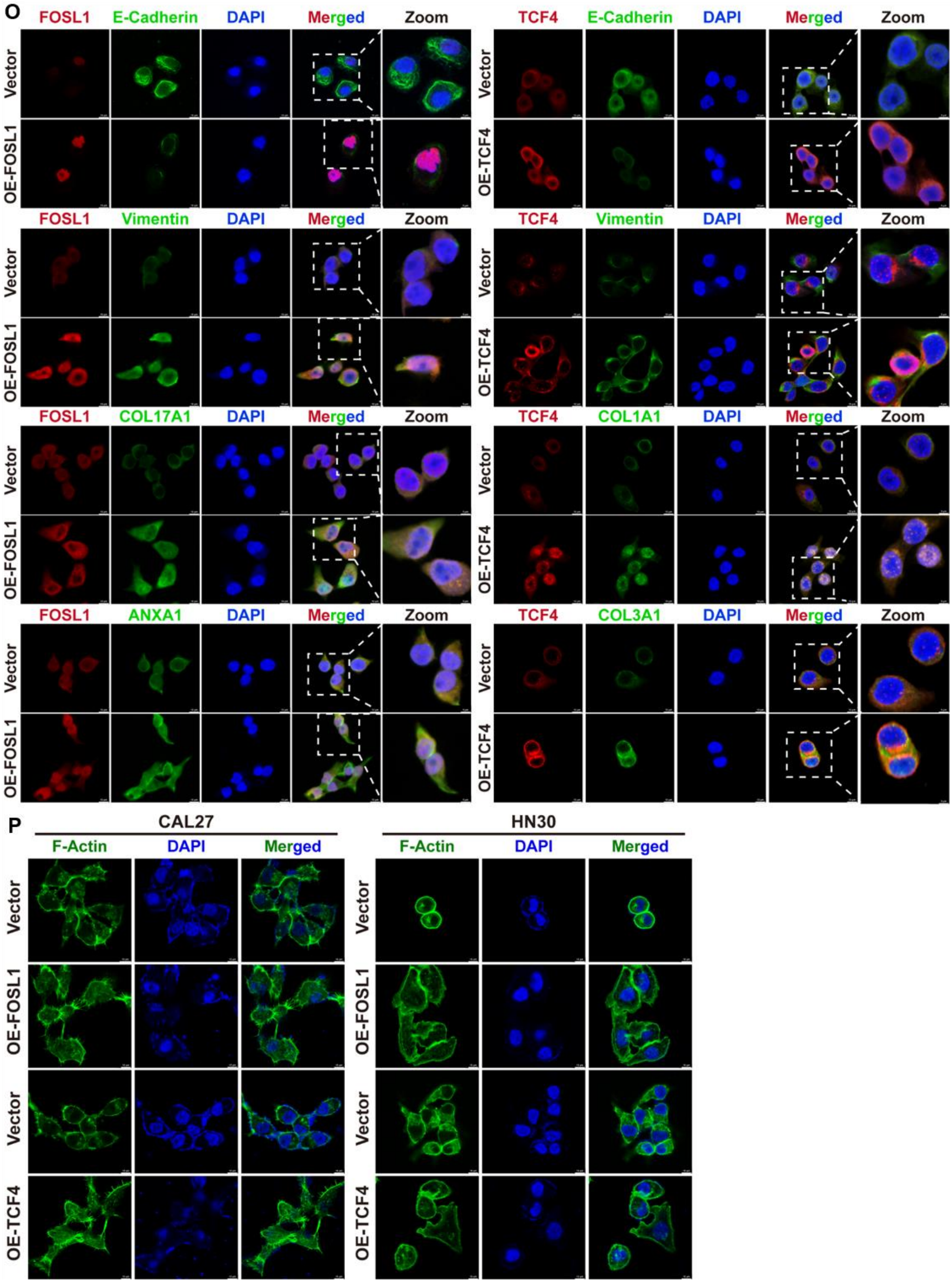
O
FOSL1
E-Cadherin
DAPI
Merged
Zoom
TCF4
Vimentin
COL17A1
COL1A1
ANXA1
COL3A1
Vector
OE-FOSL1
OE-TCF4
P
CAL27
HN30
F-Actin

(A) Schematic diagram of the recognition strategy of malignant epithelial cells. Spots both located in C1-C7 (Left, UMAP plot) and in the malignant epithelial regions (Right, including Adjacent Epithelium, ISC, and Tumor regions) were extracted for further pseudotime analysis.
(B) Trajectory plots showing the distribution of malignant epithelial cells in the pseudotime trajectory split by cluster.
(C) The dynamic expression of pseudotime-specific genes in each branch.
(D) Violin plots showing the expression of 8 subpopulations (6 epithelial subpopulations and 2 CAF subpopulations) in each cluster.
(E) Heatmap of the transcriptomic correlation among each cell subpopulation. pEMT and CAF1 showed a more similar expression pattern.
(F) Dynamically characterizing the expression profiles of representative genes across seven epithelial cell subtypes along the pseudotime axis revealed distinct patterns. Notably, genes representing Stress, early differentiation (Diff1), late differentiation (Diff2), and Hypoxia subpopulations exhibited significant expression during the early stages of malignant progression. In contrast, representative genes associated with cycling, pEMT, and CAF1 showed heightened expression in the terminal stage. It's worth mentioning that CAF2 was excluded from the analysis as it predominantly resides in the Stroma Subtypes (C10).
(G) Spots representing pEMT, CAF1 and CAF2 cells were extracted for PCA clustering analysis. PCA plot showed the differences among pEMT, CAF1 and CAF2 subpopulations.
(H) Enriched GO: BP terms for pEMT, CAF1 and CAF2 cells. BP: biological process.
(I) PCA showing the expression feature of marker genes in pEMT, CAF1, and CAF2 subpopulations.
(J) Heatmap showing the expression features of TFs among the three cells in EMT-CAF trajectory.
(K) Ridge plots of the expression of top 5 TFs in the pEMT, CAF1, and CAF2 subpopulations.
(L) Violin plots of the expression of top 5 TFs in the pEMT, CAF1, and CAF2 subpopulations.
(M) Bar chart revealing the expression level of the epithelial marker E-Cadherin, the mesenchymal marker Vimentin, and the downstream gene of TFs (FOSL1: COL17A1, ANXA1; TCF4: COL1A1, COL3A1) in CAL27 and HN30 cells transfected with FOSL1 (Left) or TCF4 (Right) plasmids. Statistical analysis was performed by student's *t* test. A *P* value < 0.05 was considered as statistical significance. *: $P < 0.05$, **: $P < 0.01$, ***: $P < 0.001$, ****: $P < 0.0001$.
(N) Immunofluorescence analysis elucidating the expression patterns of the epithelial marker E-Cadherin, the mesenchymal marker Vimentin, and the downstream gene of TFs (FOSL1: COL17A1; TCF4: COL1A1) in CAL27 cells transfected with FOSL1 or TCF4 plasmids. Red: TF (FOSL1 or TCF4); green: epithelial/mesenchymal markers or downstream genes; blue: DAPI for nucleus. Zoomed images, obtained through confocal microscopy at a 2x magnification, offer detailed views. Histopathological areas are presented at a 630× magnification with scales of 10 μm for red, blue, green, and merged images, and 5 μm for zoomed images.
(O) Immunofluorescence revealing the expression patterns of epithelial marker E-Cadherin, mesenchymal marker Vimentin, and the downstream genes of TFs (FOSL1: COL17A1, ANXA1; TCF4: COL1A1, COL3A1) in HN30 transfected with FOSL1 or TCF4 plasmids, respectively. Red: TF (FOSL1 or TCF4); green: epithelial/mesenchymal markers or downstream genes; blue: DAPI for nucleus. Zoomed images, obtained through confocal microscopy at a 2x magnification, offer detailed views. Histopathological areas are presented at a 630× magnification with scales of 10 μm for red, blue, green, and merged images, and 5 μm for zoomed images.
(P) Phalloidin staining revealing the expression characteristics of F-Actin in CAL27 and HN30 transfected with FOSL1 and TCF4 plasmids. Green: F-Actin; blue: DAPI for nucleus. The 630× magnification images of the relevant histopathological areas, with scales of 10 μm, are presented.

Figure S5 | Spatial Landscape and Interaction of Infiltrated Immune Cells in OSF-derived OSCC

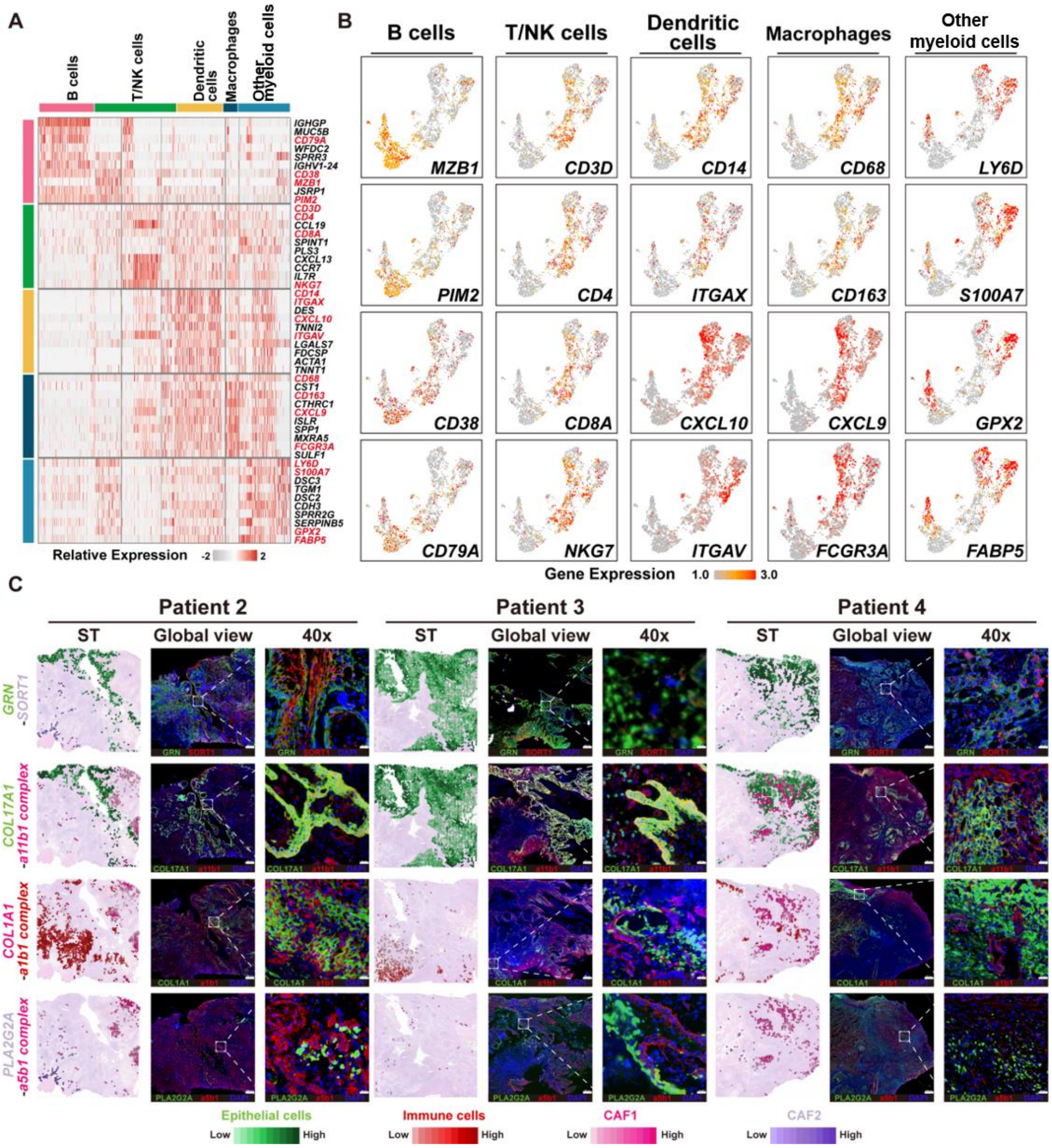

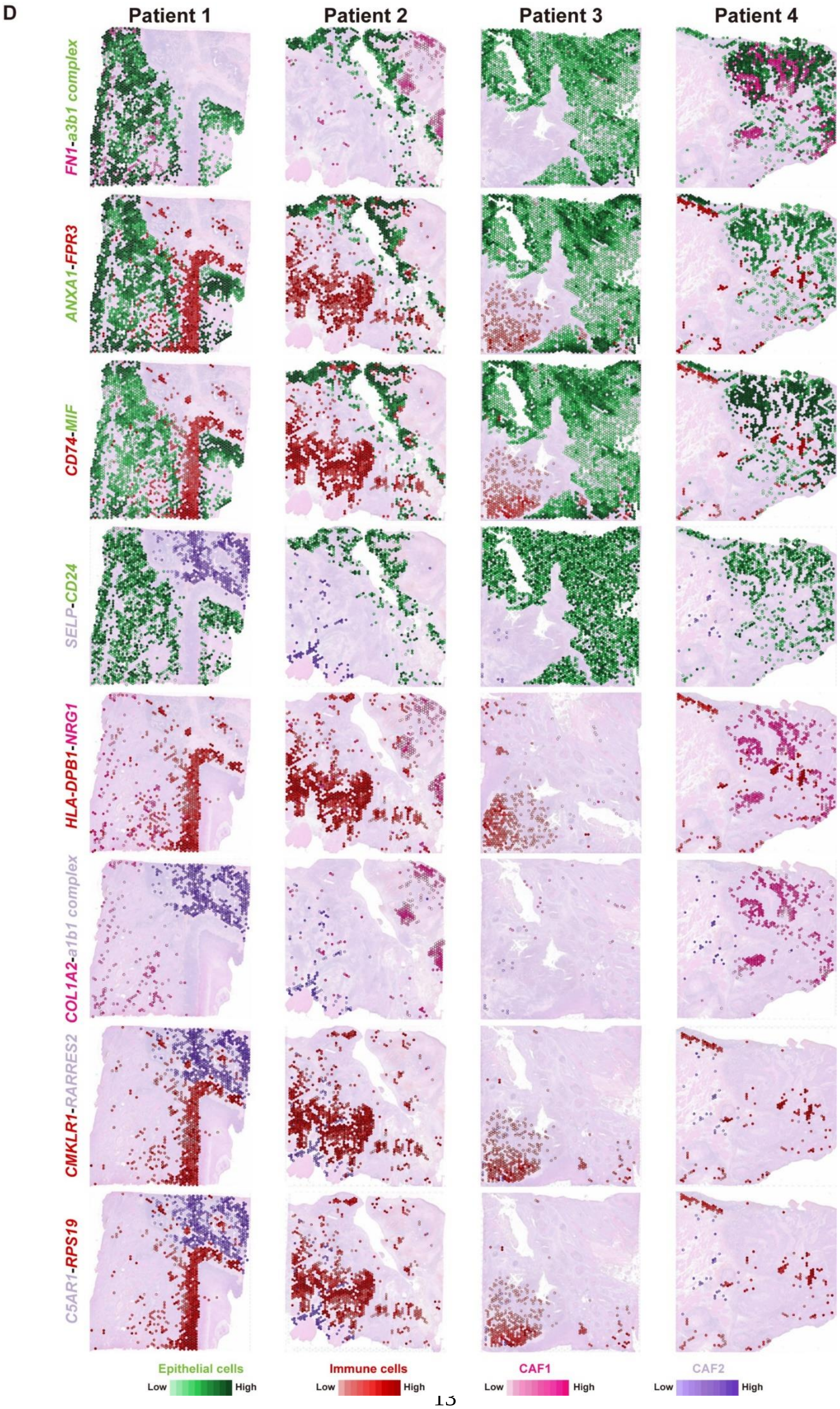
D
Patient 1
Patient 2
Patient 3
Patient 4
FN1-a3b1 complex
ANXA1-FPR3
CD74-MIF
SELP-CD24
HLA-DPB1-NRG1
COL1A2-a1b1 complex
CMKLR1-RARRES2
C5AR1-RPS19
Epithelial cells
Low
High
Immune cells
Low
High
CAF1
Low
High
CAF2
Low
High

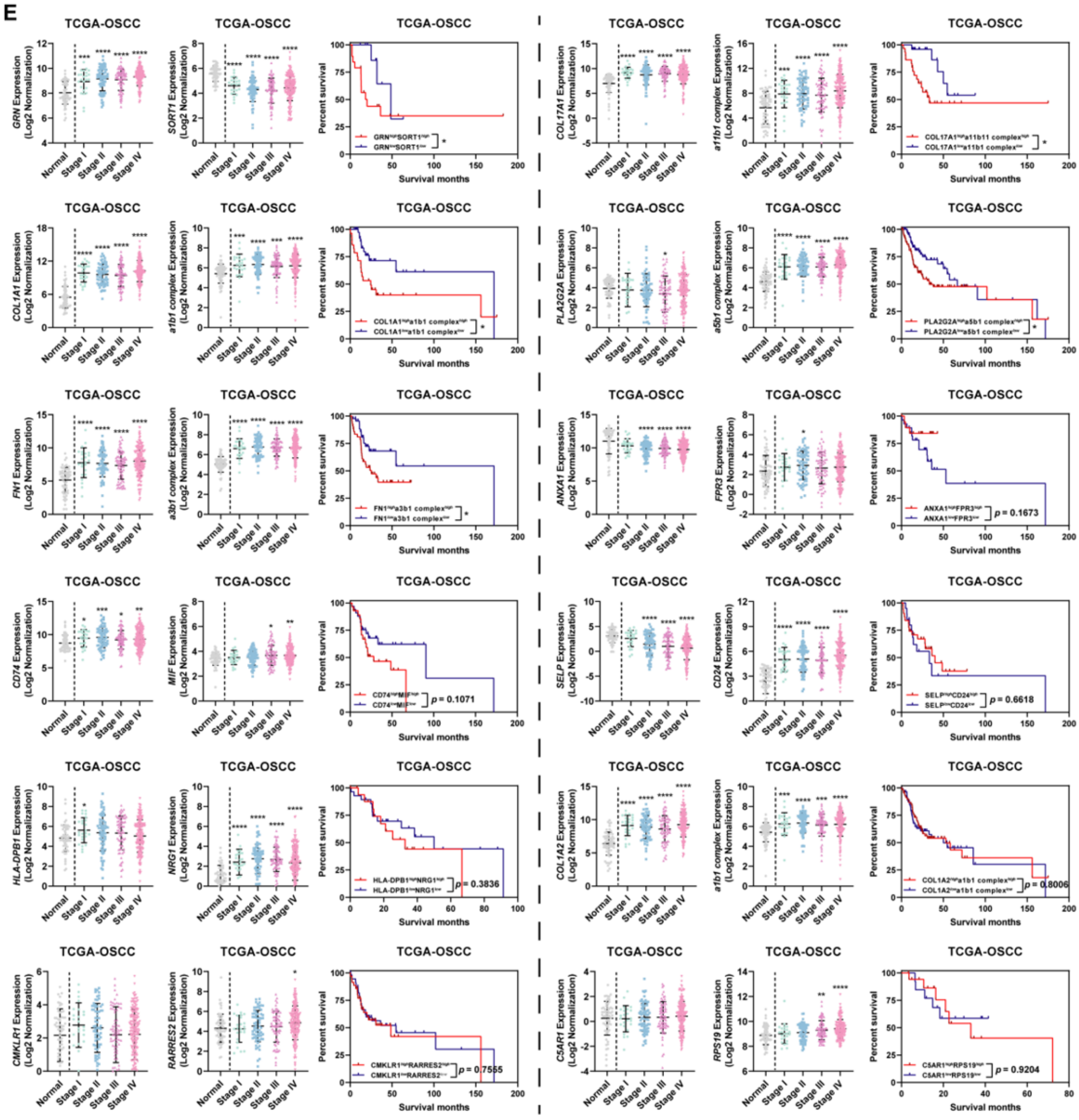
E
TCGA-OSCC
Percent survival
Survival months
Normal
Stage I
Stage II
Stage III
Stage IV
GRN Expression (Log2 Normalization)
SORT1 Expression (Log2 Normalization)
COL17A1 Expression (Log2 Normalization)
a11b1 complex Expression (Log2 Normalization)
COL1A1 Expression (Log2 Normalization)
a1b1 complex Expression (Log2 Normalization)
PLA2G2A Expression (Log2 Normalization)
a5b1 complex Expression (Log2 Normalization)
FN1 Expression (Log2 Normalization)
a3b1 complex Expression (Log2 Normalization)
ANXA1 Expression (Log2 Normalization)
FPR3 Expression (Log2 Normalization)
CD74 Expression (Log2 Normalization)
MIF Expression (Log2 Normalization)
SELP Expression (Log2 Normalization)
CD24 Expression (Log2 Normalization)
HLA-DPB1 Expression (Log2 Normalization)
NRG1 Expression (Log2 Normalization)
COL1A2 Expression (Log2 Normalization)
CMKLR1 Expression (Log2 Normalization)
RARRES2 Expression (Log2 Normalization)
C5AR1 Expression (Log2 Normalization)
RPS19 Expression (Log2 Normalization)
p = 0.1673
p = 0.1071
p = 0.6618
p = 0.3836
p = 0.8006
p = 0.7555
p = 0.9204

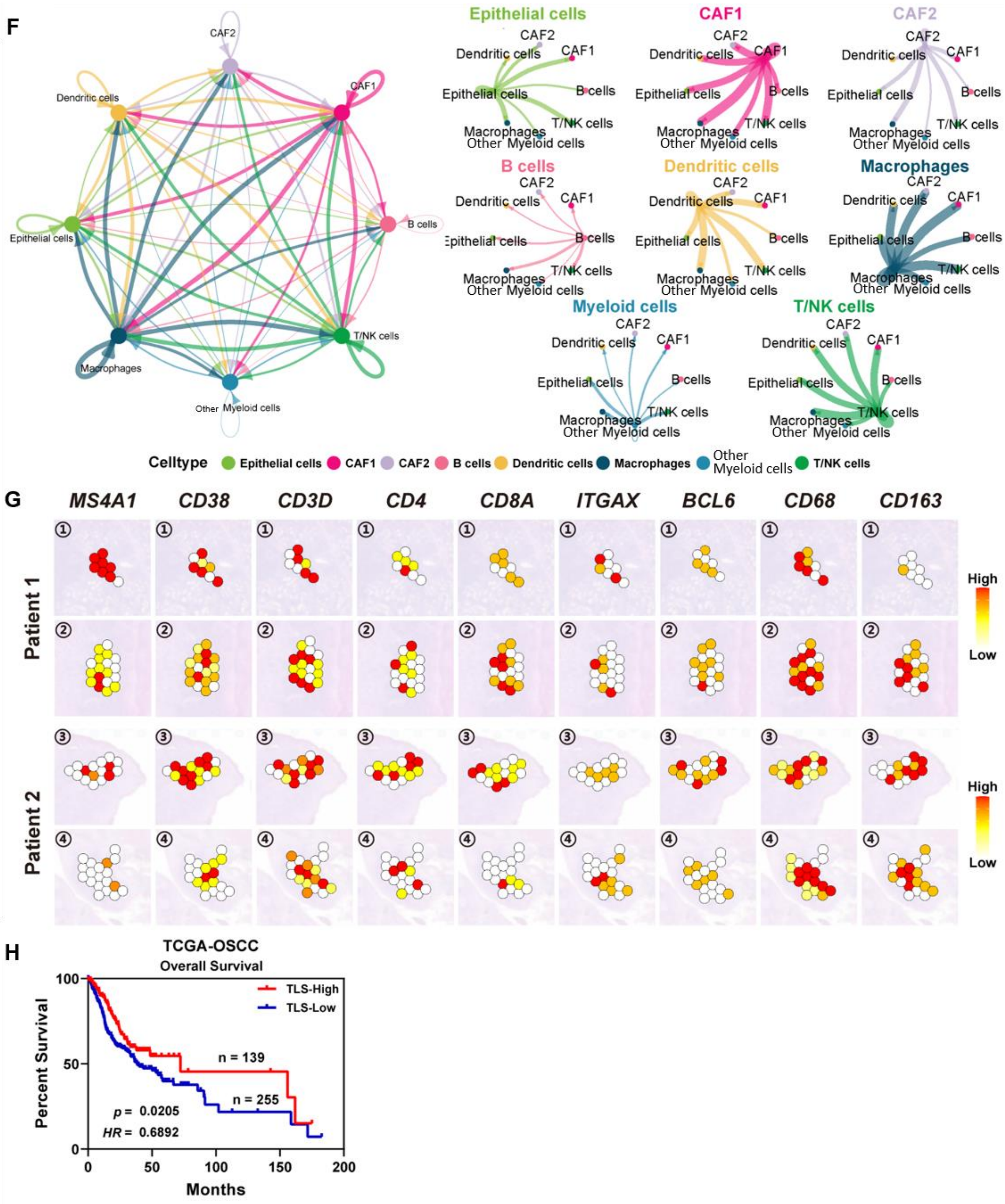
F
Epithelial cells
CAF1
CAF2
B cells
Dendritic cells
Macrophages
Myeloid cells
T/NK cells
Other Myeloid cells
Celltype
G
MS4A1
CD38
CD3D
CD4
CD8A
ITGAX
BCL6
CD68
CD163
Patient 1
Patient 2
High
Low
H
TCGA-OSCC
Overall Survival
TLS-High
TLS-Low
n = 139
n = 255
p = 0.0205
HR = 0.6892
Percent Survival
Months

(A) Heatmap showing the expression of top 10 marker genes in the 5 immune cell subpopulations.

(B) UMAP plots for the characteristics of representative genes in the 5 immune cell subpopulations.

(C) ST plots and immunofluorescence plots demonstrating the expression of representative ligand-receptor pairs in OSF-derived OSCC samples from P2-P4. Each sample contains an ST plot showing the expression pattern of two intercellular ligand-receptor pairs (left), an overall plot of multiple immunofluorescence staining of this sample (center), and an enlarged view of a representative region (right). Cell types in the ST plot include epithelial cells (green), immune cells (red), CAF1 cells (pink), and CAF2 cells (light purple). For immunofluorescence, ligand molecules (e.g., GRN, COL17A1, COL1A1, PLA2G2A) are labeled green, receptor molecules (e.g., SORT1, a11b1 complex, a1b1 complex, a5b1 complex) are labeled red, and the nuclei are labeled blue with DAPI . The immunofluorescence (middle) has a resolution of 500 μm, while the magnified view (right) is 400× with a resolution of 50 μm.

(D) ST plot demonstrating the expression patterns of the remaining 8 representative ligand-receptor pairs in 4 OSF-derived OSCC samples. Cell types in the ST plots include epithelial cells (green), immune cells (red), CAF1 cells (pink), and CAF2 cells (light purple).

(E) The clinical significances of 12 ligand-receptor pairs in OSCC cohort of TCGA. The correlation between transcriptomic level and clinical stage (left, middle) and prognosis (right) were presented. Statistical analysis was performed by student's *t* test. A P value < 0.05 was considered as statistical significance. *: $P < 0.05$, **: $P < 0.01$, ***: $P < 0.001$, ****: $P < 0.0001$

(F) Interaction network plot exhibiting the interacting patterns among 8 types of cells (log2 mean >1, P value < 0.001). The thickness of lines refers to the number of ligand-receptor pairs.

(G) ST feature plots exhibiting the expression of immune-associated marker genes in the TLS-like regions (①-④) of P1 and P2.

(H) Kaplan-Meier curve plot showing the relationship between the prognosis and TLS-signature gene set expression in the OSCC patients (n = 394) downloaded from TCGA. Patients with higher score of TLS-signature expression (TLS-high, indicating the existence of TLS-like regions in the OSCC tissues) showed a better overall survival.

Figure S6 | SM Features of OSF-derived OSCC

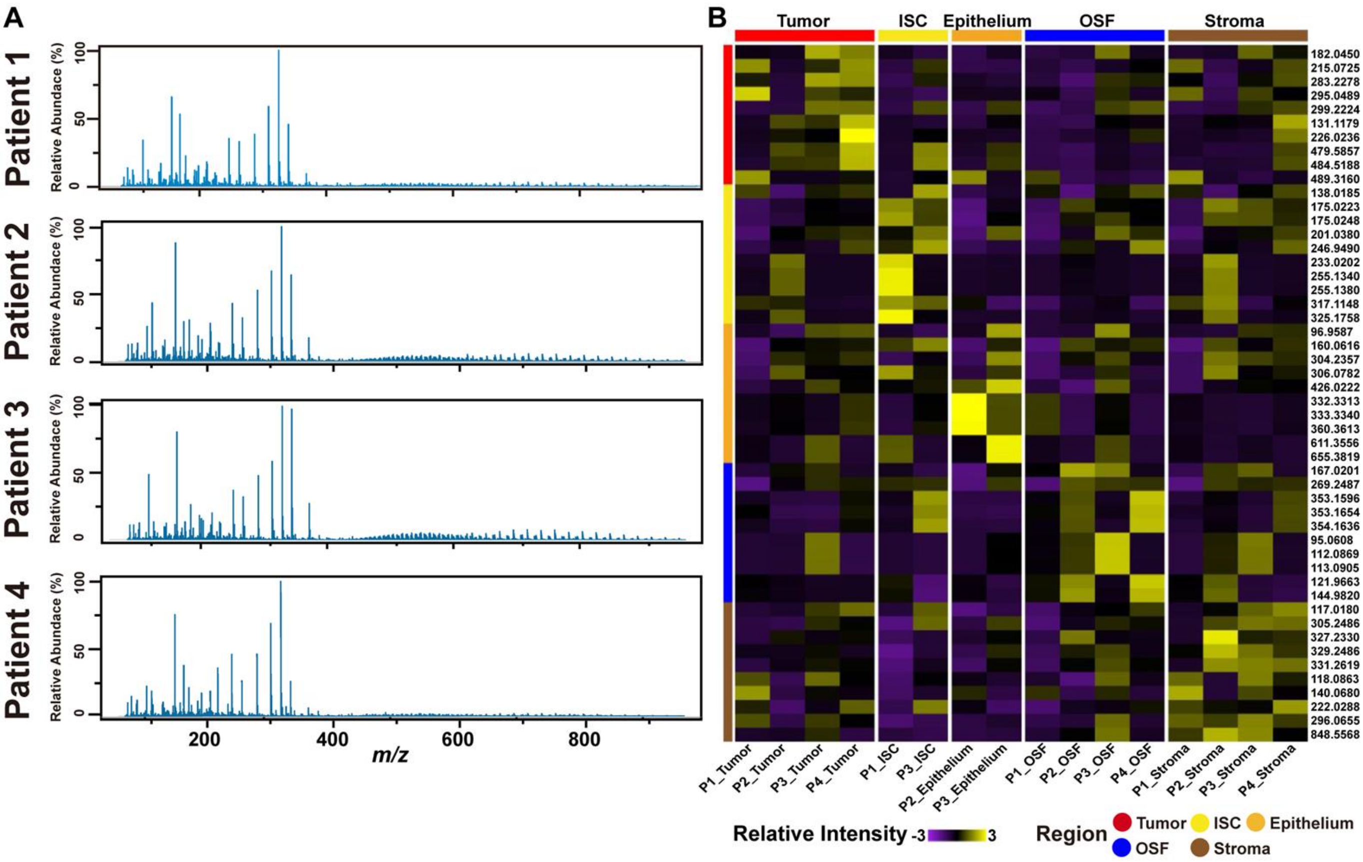

(A) Mass spectrum images revealing the average abundance of mass spectrum in the 4 samples detected by AFADESI-MSI.

(B) Heatmap displaying the abundance of top 10 m/z metabolites in each histopathological region of 4 samples.

Figure S7 | Distribution of Differential Metabolites in OSF-derived OSCC in Global Metabolic Flux

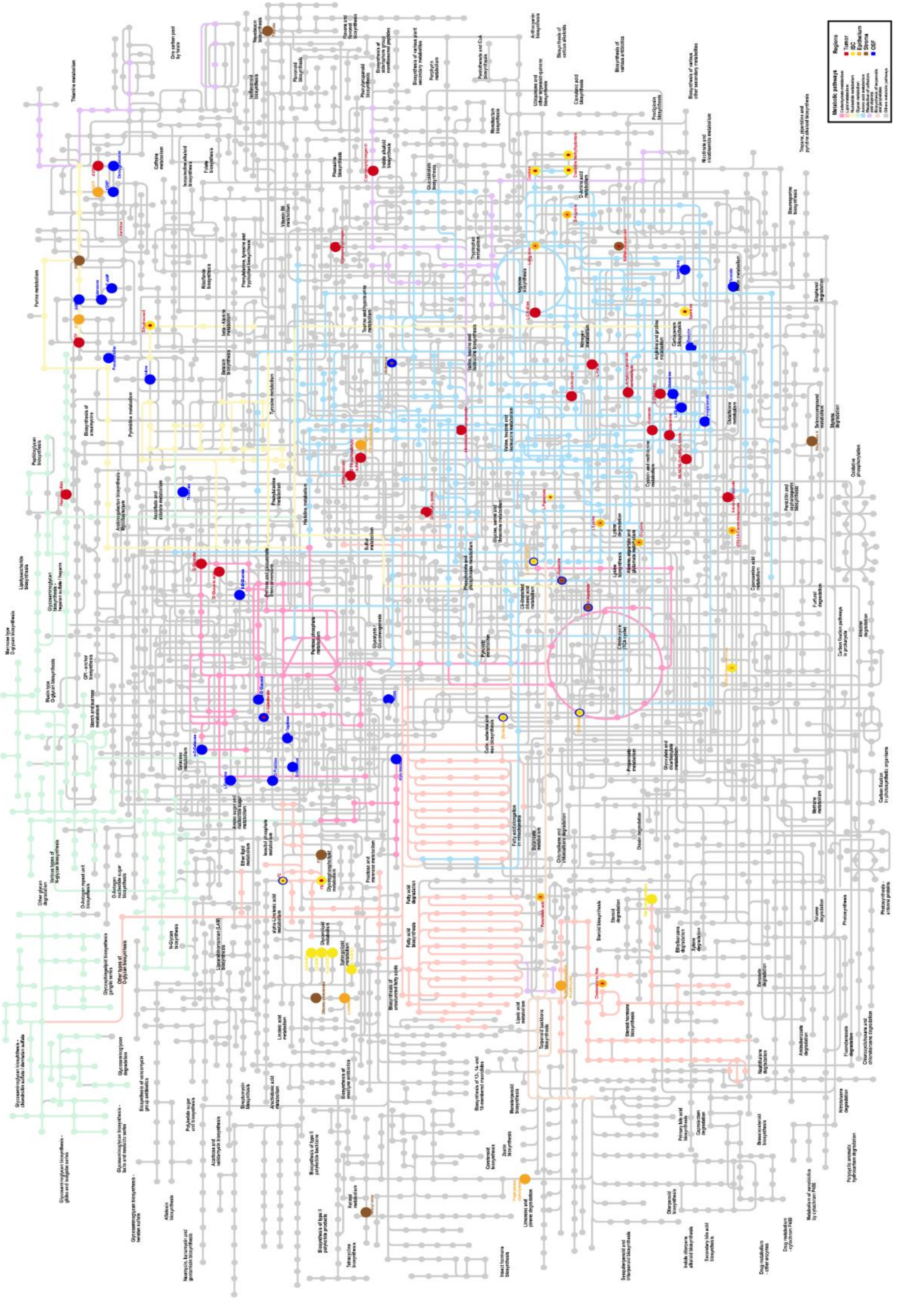

Figure S8 | Abundance and Distribution of Metabolites Associated with Carbohydrate and Lipid Metabolism in OSF-derived OSCC

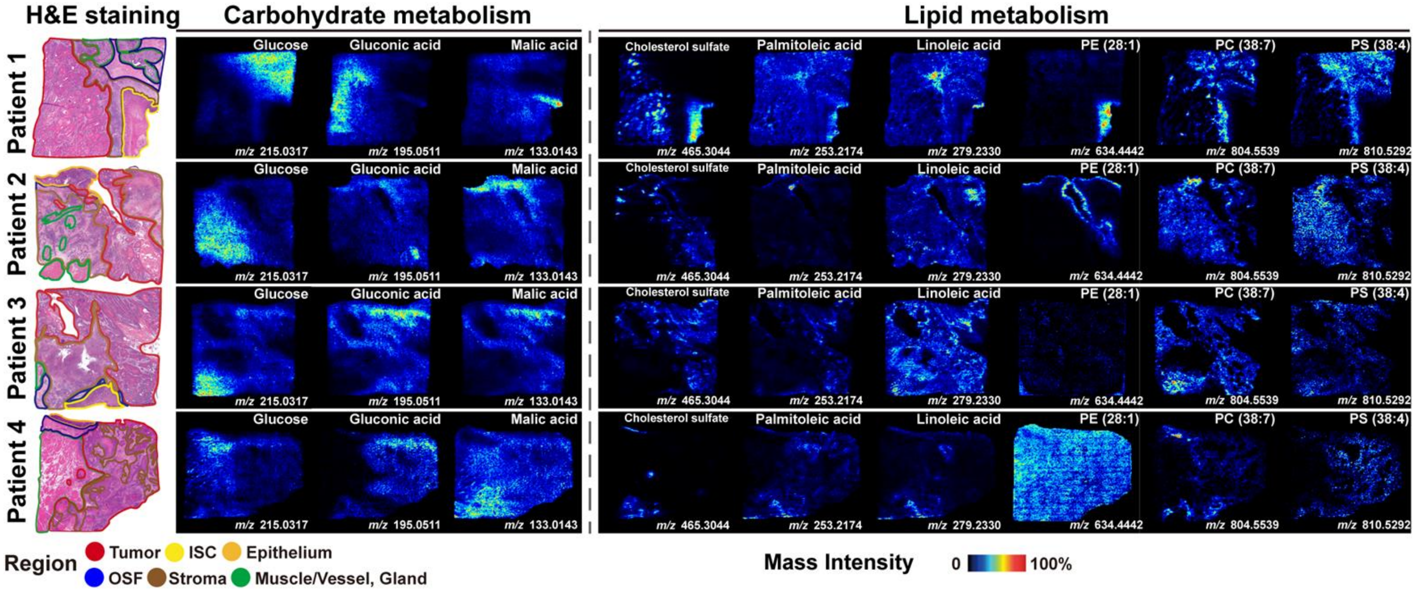

Figure S9 | Polyamine Metabolism Represents as the Hallmark of Amino Acid Metabolic Reprogramming in OSF-derived OSCC

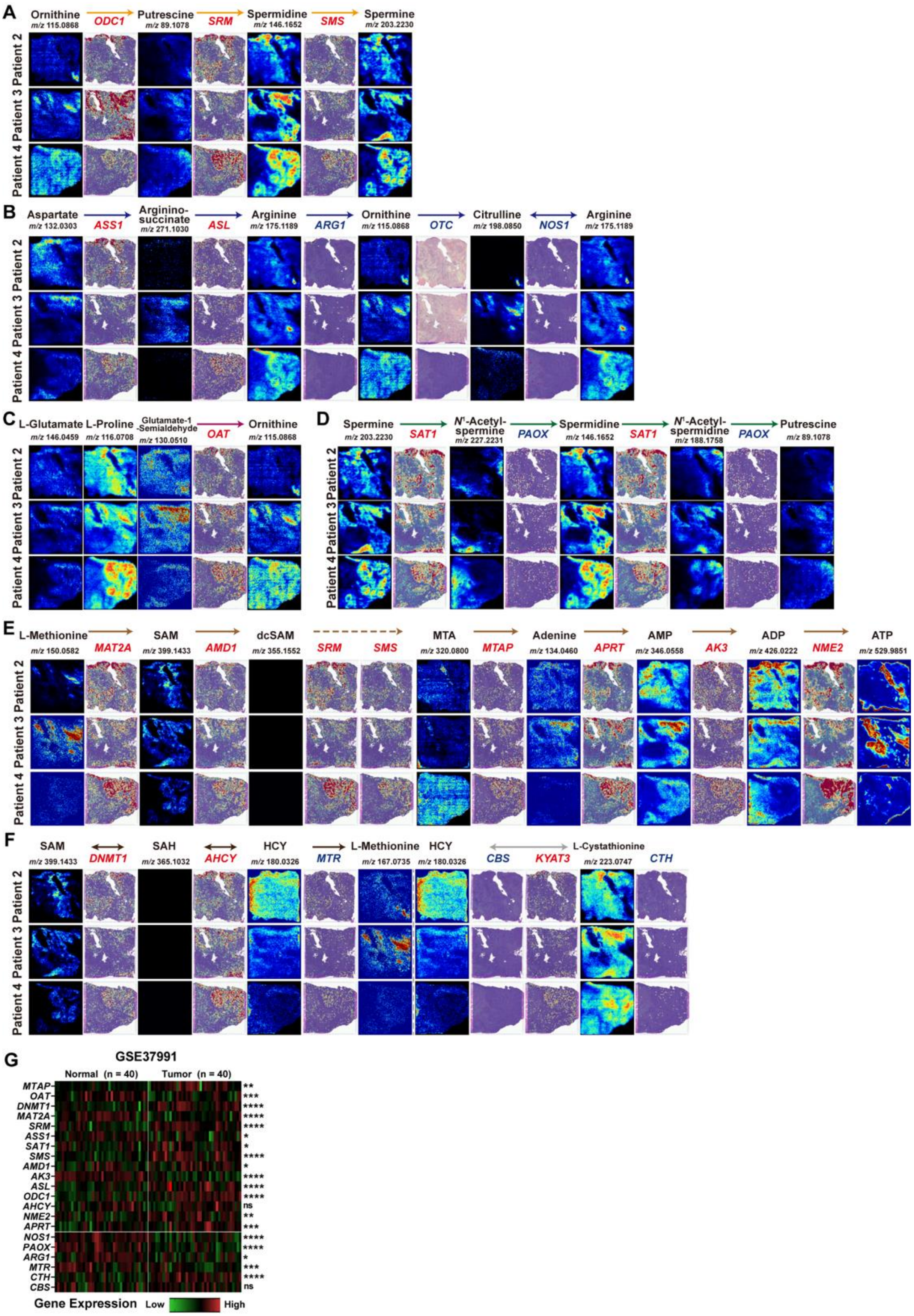

(A) The spatial abundance or expression of essential metabolites and related enzymes in the polyamine synthetic metabolism (yellow arrows in Fig. 5A) of P2-P4.

(B) The spatial distribution feature of metabolic products and enzymes in the urea cycle (blue arrows in Fig. 5A) of P2-P4.
(C) The ST and SM feature of ornithine salvage synthesis (purple arrow in Fig. 5A) in P2-P4.
(D) The spatial multi-omic feature of polyamine catabolic metabolism (green arrows in Fig. 5A) in P2-P4.
(E) The spatial multi-omic characteristics of methionine salvage metabolism (SAM-dcSAM pathway; light brown arrows in Fig. 5A) in P2-P4.
(F) The spatial multi-omic characteristics of methionine salvage synthesis (SAM-SAH-HCY pathway; dark brown arrows in Fig. 5A) and cysteine salvage synthesis (grey arrows in Fig. 5A) in P2-P4.
(G) Heatmap showing the transcriptomic expression level of PM enzymes in betel nut-associated OSCC (GSE37991). The statistical significance was presented based on two-tail student's *t* test. *: $P < 0.05$, **: $P < 0.01$, ***: $P < 0.001$, ****: $P < 0.0001$

Table S1 | Characteristics of Four OSF-derived OSCC Patients Included in this Study

Patient	Age	Gender	Alcohol	Betel Quid	Cigarette	OSF	BQ Chewing Frequency (Pieces per day, years)	Maximal Mouth Opening (cm)	Perineural Invasion	Tumor Position	Tumor Size (cm)	TNM	Clinical Stage
P1	56	M	√	√	√	√	15, 10	3.0	√	Buccal	2.0*2.0*1.5	T3N0M0	III
P2	49	M		√	√	√	15, 15	2.5		Gingiva	3.5*2.5*1.0	T3N0M0	III
P3	58	F	√	√	√	√	6, 5	3.0		Gingiva	1.0*1.0*1.0	T3N0M0	III
P4	57	M	√	√	√	√	30, 13	2.0	√	Tongue	4.0*3.0*2.5	T3N1M0	III

Table S2 | Statistics of 10x Genomics Visium Spatial Transcriptomics

Patient	Mean nUMI	Mean nGene	Mean mito percent*	Mean Reads per Spot	Total spots
P1	18052.514	3830.954	0.027	79,845	4199
P2	11836.968	3128.328	0.027	92,218	4290
P3	8426.682	2252.173	0.031	63,534	4514
P4	12088.332	3013.732	0.056	69,246	4316

* Mean mito percent: Mean percentage of mitochondrial genes expressed in each spot.

Table S3 | Differentially Expressed Genes among pEMT, CAF1, and CAF2

Genes	*P* value	avg_log2FC	pct.1	pct.2	FDR	Group
MMP1	7.48E-155	3.805	0.973	0.549	1.76E-150	CAF1
MMP3	1.56E-51	3.458	0.629	0.299	3.68E-47	CAF1
COL3A1	1.56E-207	2.485	1	0.834	3.69E-203	CAF1
POSTN	3.25E-180	2.451	0.97	0.575	7.66E-176	CAF1
FN1	2.15E-166	2.291	0.993	0.759	5.07E-162	CAF1
MMP11	1.59E-122	2.178	0.784	0.264	3.75E-118	CAF1
COL1A2	1.07E-208	2.151	1	0.947	2.53E-204	CAF1
MMP13	5.95E-90	2.146	0.622	0.158	1.40E-85	CAF1
COL1A1	2.08E-195	2.095	1	0.912	4.92E-191	CAF1
SPARC	5.60E-213	2.066	1	0.920	1.32E-208	CAF1
COL5A2	8.34E-194	1.958	0.984	0.603	1.97E-189	CAF1
LUM	4.87E-187	1.917	0.995	0.823	1.15E-182	CAF1
CST1	3.06E-61	1.809	0.565	0.195	7.22E-57	CAF1
COL5A1	3.26E-178	1.754	0.973	0.599	7.69E-174	CAF1
COL12A1	2.69E-158	1.719	0.921	0.508	6.35E-154	CAF1

COL6A1	2.65E-172	1.682	1	0.912	6.25E-168	CAF1
ISLR	6.51E-110	1.442	0.881	0.568	1.54E-105	CAF1
MXRA5	2.37E-115	1.377	0.838	0.399	5.60E-111	CAF1
THBS2	8.54E-97	1.322	0.874	0.593	2.01E-92	CAF1
ASPN	1.50E-82	1.279	0.634	0.198	3.53E-78	CAF1
CTHRC1	2.35E-89	1.269	0.809	0.447	5.55E-85	CAF1
MMP14	4.88E-62	1.218	0.853	0.613	1.15E-57	CAF1
COL6A2	6.71E-123	1.196	1	0.986	1.58E-118	CAF1
FSTL1	6.88E-118	1.188	0.979	0.819	1.62E-113	CAF1
COL11A1	7.75E-86	1.165	0.552	0.114	1.83E-81	CAF1
WNT5A	1.00E-79	1.118	0.676	0.224	2.36E-75	CAF1
LOXL2	2.24E-89	1.095	0.735	0.302	5.29E-85	CAF1
SULF1	6.36E-85	1.094	0.803	0.452	1.50E-80	CAF1
COL7A1	2.77E-78	1.083	0.868	0.487	6.52E-74	CAF1
COL6A3	5.48E-104	1.081	0.991	0.859	1.29E-99	CAF1
BGN	8.87E-106	1.065	0.990	0.899	2.09E-101	CAF1
COL16A1	1.21E-91	1.050	0.887	0.564	2.84E-87	CAF1
COL5A3	4.12E-79	1.048	0.782	0.377	9.71E-75	CAF1
MMP9	3.54E-40	1.039	0.559	0.251	8.34E-36	CAF1
ADAMTS2	2.94E-83	1.016	0.755	0.376	6.94E-79	CAF1
GJA1	7.81E-104	1.009	0.972	0.574	1.84E-99	CAF1
KRT6C	1.01E-19	2.342	0.667	0.583	2.38E-15	pEMT
PI3	6.65E-23	2.181	0.802	0.581	1.57E-18	pEMT
SPRR1B	6.57E-14	1.647	0.601	0.562	1.55E-09	pEMT
KRT6A	1.49E-89	1.620	1	0.962	3.52E-85	pEMT
KRT16	3.01E-43	1.610	1	0.979	7.10E-39	pEMT
KRT6B	2.15E-57	1.583	1	0.968	5.06E-53	pEMT
FGFBP1	7.77E-42	1.548	0.605	0.235	1.83E-37	pEMT
KLK10	7.80E-27	1.509	0.564	0.322	1.84E-22	pEMT
KRT14	1.06E-68	1.492	1	1	2.50E-64	pEMT
CSTA	6.72E-46	1.489	0.988	0.804	1.59E-41	pEMT
KRT5	6.41E-68	1.453	1	0.957	1.51E-63	pEMT
SFN	9.80E-60	1.452	1	0.932	2.31E-55	pEMT
TGM1	5.55E-32	1.435	0.477	0.180	1.31E-27	pEMT
SPRR2A	4.22E-11	1.431	0.580	0.515	9.94E-07	pEMT
DSP	1.14E-74	1.415	0.996	0.675	2.69E-70	pEMT
RHCG	3.20E-11	1.382	0.494	0.363	7.55E-07	pEMT
PERP	1.15E-86	1.365	1	0.894	2.72E-82	pEMT
S100A8	2.52E-59	1.360	1	0.985	5.95E-55	pEMT
TRIM29	6.09E-43	1.318	0.909	0.625	1.44E-38	pEMT
IGFBP6	2.28E-12	1.286	0.634	0.648	5.38E-08	pEMT
SPRR2E	1.87E-09	1.258	0.514	0.408	4.40E-05	pEMT
S100A14	7.24E-63	1.242	0.951	0.613	1.71E-58	pEMT
COL17A1	3.97E-45	1.228	0.893	0.513	9.36E-41	pEMT
DSG3	2.54E-60	1.227	0.934	0.498	5.99E-56	pEMT
SLC2A1	1.98E-34	1.216	0.724	0.414	4.67E-30	pEMT
GJB2	1.76E-50	1.192	0.918	0.519	4.16E-46	pEMT
TUBA1C	2.46E-77	1.185	0.996	0.783	5.80E-73	pEMT
TUBA4A	1.86E-46	1.184	0.901	0.567	4.38E-42	pEMT
ATP1B3	1.28E-42	1.179	0.951	0.685	3.01E-38	pEMT
FABP5	5.37E-65	1.170	1	0.916	1.27E-60	pEMT
S100A16	2.28E-60	1.156	1	0.841	5.38E-56	pEMT
SERPINB5	1.21E-66	1.143	0.905	0.408	2.84E-62	pEMT
S100A2	7.16E-49	1.137	1	0.933	1.69E-44	pEMT
SLC7A5	7.57E-36	1.130	0.765	0.454	1.79E-31	pEMT
PKP3	1.75E-44	1.118	0.815	0.447	4.14E-40	pEMT
PKP1	8.56E-60	1.113	0.984	0.656	2.02E-55	pEMT
PPIF	6.34E-46	1.088	0.811	0.413	1.50E-41	pEMT
LAMC2	7.39E-40	1.079	0.872	0.581	1.74E-35	pEMT
IL36G	6.31E-51	1.063	0.395	0.069	1.49E-46	pEMT
SPRR2D	5.94E-12	1.053	0.535	0.376	1.40E-07	pEMT
KRT17	2.29E-47	1.047	1	0.975	5.41E-43	pEMT

JUP	1.61E-51	1.045	1	0.814	3.79E-47	pEMT
KLF5	2.17E-38	1.043	0.695	0.336	5.13E-34	pEMT
UPK3BL1	3.02E-13	1.032	0.539	0.391	7.13E-09	pEMT
CTSC	5.61E-13	1.007	0.914	0.797	1.32E-08	pEMT
S100A9	5.26E-44	1.003	1	0.994	1.24E-39	pEMT
LAD1	1.61E-55	1.003	0.877	0.437	3.79E-51	pEMT
S100A7	7.93E-27	1.002	0.893	0.685	1.87E-22	pEMT
MUC5B	5.25E-177	6.586	0.750	0.125	1.24E-172	CAF2
BPIFB2	4.44E-194	4.480	0.729	0.056	1.05E-189	CAF2
TFF3	4.51E-202	4.350	0.770	0.076	1.06E-197	CAF2
AZGP1	3.04E-210	4.203	0.750	0.044	7.17E-206	CAF2
WFDC2	4.21E-175	3.877	0.765	0.147	9.93E-171	CAF2
IGHA1	2.63E-185	3.702	0.987	0.583	6.19E-181	CAF2
ZG16B	1.85E-201	3.336	0.712	0.030	4.37E-197	CAF2
IGHM	4.86E-180	3.301	0.826	0.162	1.15E-175	CAF2
PLA2G2A	3.37E-259	3.064	0.861	0.036	7.95E-255	CAF2
PIGR	7.76E-179	3.032	0.669	0.039	1.83E-174	CAF2
LTF	6.82E-181	2.890	0.667	0.033	1.61E-176	CAF2
LCN2	2.37E-153	2.855	0.761	0.172	5.59E-149	CAF2
IGLC2	6.90E-172	2.835	0.996	0.647	1.63E-167	CAF2
CLU	2.06E-236	2.833	0.96	0.270	4.85E-232	CAF2
IGLC3	6.36E-149	2.780	0.971	0.495	1.50E-144	CAF2
BPIFB1	1.06E-132	2.777	0.506	0.018	2.51E-128	CAF2
IGHG2	3.75E-177	2.780	0.980	0.508	8.84E-173	CAF2
IGHG1	3.80E-160	2.704	0.998	0.673	8.95E-156	CAF2
IGLC1	3.84E-173	2.685	0.993	0.531	9.06E-169	CAF2
SAA2	2.20E-120	2.632	0.700	0.199	5.19E-116	CAF2
JCHAIN	7.18E-146	2.627	0.917	0.342	1.69E-141	CAF2
C3	4.63E-196	2.601	0.987	0.624	1.09E-191	CAF2
APOD	1.76E-177	2.583	0.863	0.212	4.15E-173	CAF2
IGKC	6.82E-153	2.399	0.998	0.829	1.61E-148	CAF2
IGHG3	9.35E-141	2.375	0.991	0.588	2.21E-136	CAF2
CRISP3	7.06E-133	2.351	0.492	0.011	1.67E-128	CAF2
SAA1	9.12E-57	2.332	0.861	0.675	2.15E-52	CAF2
CFD	1.00E-214	2.326	0.897	0.176	2.36E-210	CAF2
AGR2	8.54E-105	2.285	0.474	0.045	2.02E-100	CAF2
IGHG4	9.14E-149	2.264	0.996	0.746	2.15E-144	CAF2
PLTP	3.09E-146	2.225	0.870	0.393	7.28E-142	CAF2
FAM3D	1.15E-172	2.209	0.627	0.022	2.71E-168	CAF2
GPX3	2.14E-170	2.174	0.958	0.478	5.06E-166	CAF2
CCN5	1.32E-201	2.159	0.785	0.082	3.12E-197	CAF2
FDCSP	2.15E-45	2.115	0.456	0.167	5.08E-41	CAF2
KRT7	1.59E-144	2.113	0.658	0.097	3.74E-140	CAF2
MZB1	1.09E-160	2.105	0.895	0.308	2.57E-156	CAF2
IGFBP4	7.46E-159	2.089	0.993	0.963	1.76E-154	CAF2
KRT13	7.04E-72	1.959	0.812	0.457	1.66E-67	CAF2
PTGDS	8.94E-173	1.948	0.857	0.203	2.11E-168	CAF2
KRT19	5.76E-144	1.838	0.700	0.116	1.36E-139	CAF2
PLAC9	1.83E-186	1.811	0.854	0.204	4.32E-182	CAF2
RNASE1	4.64E-121	1.778	0.919	0.618	1.10E-116	CAF2
SSR4	9.93E-170	1.770	0.996	0.939	2.34E-165	CAF2
TNXB	1.13E-173	1.765	0.763	0.115	2.68E-169	CAF2
MGP	1.10E-120	1.763	0.919	0.526	2.60E-116	CAF2
SCGB3A1	4.90E-143	1.724	0.532	0.015	1.16E-138	CAF2
SLPI	1.62E-50	1.682	0.886	0.835	3.82E-46	CAF2
TIMP1	2.79E-142	1.673	0.998	0.965	6.57E-138	CAF2
DEFB1	2.55E-112	1.583	0.608	0.113	6.00E-108	CAF2
PI16	6.55E-155	1.539	0.546	0.005	1.54E-150	CAF2
ADIRF	1.04E-125	1.536	0.902	0.419	2.45E-121	CAF2
CCL18	6.87E-64	1.512	0.702	0.359	1.62E-59	CAF2
C1QA	1.21E-125	1.504	0.967	0.787	2.86E-121	CAF2
FCGRT	8.75E-136	1.487	0.913	0.497	2.06E-131	CAF2

AQP5	1.54E-134	1.474	0.488	0.007	3.63E-130	CAF2
SOD3	6.37E-138	1.429	0.834	0.282	1.50E-133	CAF2
CYBA	1.04E-162	1.427	0.995	0.785	2.45E-158	CAF2
IGHGP	1.02E-110	1.417	0.664	0.132	2.40E-106	CAF2
RARRES1	2.24E-178	1.399	0.711	0.059	5.29E-174	CAF2
FTL	7.82E-117	1.382	1	1	1.85E-112	CAF2
C1QB	4.47E-111	1.346	0.98	0.781	1.05E-106	CAF2
CCL19	1.96E-134	1.340	0.644	0.075	4.62E-130	CAF2
IFITM2	1.97E-104	1.325	0.919	0.646	4.66E-100	CAF2
NPDC1	3.13E-134	1.312	0.828	0.290	7.37E-130	CAF2
F13A1	2.34E-108	1.283	0.703	0.213	5.53E-104	CAF2
CXCL12	8.33E-119	1.279	0.906	0.414	1.96E-114	CAF2
CFB	8.27E-96	1.273	0.752	0.303	1.95E-91	CAF2
TCN1	4.04E-108	1.266	0.427	0.015	9.52E-104	CAF2
CD14	3.24E-105	1.262	0.915	0.591	7.63E-101	CAF2
KLK12	2.84E-58	1.235	0.410	0.093	6.69E-54	CAF2
ACKR1	3.34E-116	1.216	0.590	0.085	7.88E-112	CAF2
A2M	1.94E-94	1.189	0.986	0.787	4.58E-90	CAF2
MUC7	1.75E-60	1.182	0.253	0.009	4.12E-56	CAF2
KRT4	2.90E-63	1.178	0.584	0.188	6.83E-59	CAF2
IGFBP5	4.46E-75	1.172	0.843	0.551	1.05E-70	CAF2
PODN	6.26E-113	1.162	0.714	0.205	1.48E-108	CAF2
LRRC26	3.32E-121	1.159	0.439	0.004	7.84E-117	CAF2
FBLN1	4.21E-86	1.157	0.966	0.797	9.92E-82	CAF2
CHRDL2	3.37E-131	1.155	0.524	0.026	7.94E-127	CAF2
SELENOP	2.76E-83	1.153	0.917	0.588	6.50E-79	CAF2
MT1M	7.94E-104	1.133	0.684	0.181	1.87E-99	CAF2
PTGIS	3.54E-144	1.133	0.559	0.025	8.35E-140	CAF2
CCDC80	6.35E-51	1.122	0.769	0.535	1.50E-46	CAF2
MSLN	2.78E-64	1.117	0.400	0.074	6.54E-60	CAF2
XBP1	7.73E-62	1.110	0.852	0.549	1.82E-57	CAF2
SERPING1	2.61E-82	1.056	0.957	0.765	6.15E-78	CAF2
FOLR2	9.36E-103	1.050	0.627	0.153	2.21E-98	CAF2
DMBT1	7.16E-82	1.040	0.313	0.004	1.69E-77	CAF2
CLDN10	4.53E-82	1.027	0.320	0.006	1.07E-77	CAF2
STAB1	1.50E-79	1.027	0.846	0.499	3.54E-75	CAF2
AQP1	1.85E-73	1.019	0.848	0.471	4.36E-69	CAF2
SPDEF	3.04E-99	1.015	0.367	0.003	7.18E-95	CAF2
GYPC	9.19E-99	1.014	0.758	0.308	2.17E-94	CAF2
CP	9.26E-130	1.013	0.505	0.020	2.18E-125	CAF2
ATP2A3	6.00E-85	1.013	0.752	0.291	1.41E-80	CAF2
LYZ	1.17E-40	1.008	0.859	0.639	2.75E-36	CAF2
C1QC	6.00E-66	1.007	0.848	0.570	1.41E-61	CAF2

Table S4 | Ligand-Receptor Pairs Significantly Expressed among Immune Cells, Epithelial Cells, and Fibroblasts (CAF1 and CAF2)

SOURCE	TARGET	count
Epithelial cells	Epithelial cells	73
Epithelial cells	CAF1	62
Epithelial cells	Dendritic cells	40
Epithelial cells	Myeloid cells	28
Epithelial cells	CAF2	41
Epithelial cells	Macrophages	67
Epithelial cells	B cells	26
Epithelial cells	T/NK cells	62
CAF1	Epithelial cells	100
CAF1	CAF1	117
CAF1	Dendritic cells	100
CAF1	Myeloid cells	67
CAF1	CAF2	103
CAF1	Macrophages	137

CAF1	B cells	65
CAF1	T/NK cells	118
Dendritic cells	Epithelial cells	81
Dendritic cells	CAF1	88
Dendritic cells	Dendritic cells	75
Dendritic cells	Myeloid cells	46
Dendritic cells	CAF2	68
Dendritic cells	Macrophages	110
Dendritic cells	B cells	40
Dendritic cells	T/NK cells	98
Myeloid cells	Epithelial cells	36
Myeloid cells	CAF1	25
Myeloid cells	Dendritic cells	16
Myeloid cells	Myeloid cells	9
Myeloid cells	CAF2	19
Myeloid cells	Macrophages	43
Myeloid cells	B cells	10
Myeloid cells	T/NK cells	43
CAF2	Epithelial cells	57
CAF2	CAF1	57
CAF2	Dendritic cells	43
CAF2	Myeloid cells	28
CAF2	CAF2	52
CAF2	Macrophages	77
CAF2	B cells	26
CAF2	T/NK cells	65
Macrophages	Epithelial cells	114
Macrophages	CAF1	134
Macrophages	Dendritic cells	126
Macrophages	Myeloid cells	90
Macrophages	CAF2	117
Macrophages	Macrophages	155
Macrophages	B cells	84
Macrophages	T/NK cells	138
B cells	Epithelial cells	22
B cells	CAF1	17
B cells	Dendritic cells	11
B cells	Myeloid cells	5
B cells	CAF2	16
B cells	Macrophages	36
B cells	B cells	6
B cells	T/NK cells	35
T/NK cells	Epithelial cells	88
T/NK cells	CAF1	104
T/NK cells	Dendritic cells	94
T/NK cells	Myeloid cells	64
T/NK cells	CAF2	84
T/NK cells	Macrophages	125
T/NK cells	B cells	56
T/NK cells	T/NK cells	120

Table S5 | TLS-DEGs of 4 TLS-like Regions in OSF-derived OSCC

Genes	*P* value	avg_log2FC	pct.1	pct.2	FDR
IGHG2	8.36E-31	3.715	1	0.691	3.06E-26
IGLC1	1.26E-24	3.398	1	0.714	4.60E-20
IGKC	6.49E-31	3.239	1	0.971	2.38E-26
IGHG1	9.53E-27	3.181	1	0.900	3.49E-22
IGHG3	2.28E-28	3.073	1	0.825	8.36E-24

IGLC3	4.32E-21	2.902	1	0.697	1.58E-16
IGLC2	5.13E-25	2.866	1	0.875	1.88E-20
IGHG4	1.03E-28	2.847	1	0.941	3.77E-24
IGHA2	1.65E-17	2.843	0.770	0.333	6.04E-13
JCHAIN	5.44E-32	2.717	0.984	0.482	1.99E-27
IGHA1	1.30E-28	2.659	1	0.810	4.77E-24
XBP1	2.44E-28	2.603	0.967	0.590	8.92E-24
IGHGP	1.52E-23	2.595	0.672	0.194	5.58E-19
SSR4	5.41E-23	2.563	1	0.839	1.98E-18
IGHM	2.69E-26	2.391	0.836	0.320	9.83E-22
HERPUD1	8.03E-25	2.289	0.934	0.583	2.94E-20
CXCR4	2.24E-40	2.274	0.934	0.336	8.20E-36
CD52	7.85E-31	2.255	0.885	0.351	2.87E-26
TXNIP	1.81E-30	2.211	0.984	0.594	6.61E-26
MZB1	4.15E-27	2.159	0.951	0.455	1.52E-22
DERL3	2.68E-34	2.011	0.902	0.311	9.82E-30
TSC22D3	3.70E-24	1.988	0.951	0.660	1.36E-19
PIM2	8.73E-35	1.953	0.820	0.252	3.20E-30
CLU	3.70E-13	1.934	0.754	0.354	1.35E-08
ISG20	6.15E-29	1.907	0.902	0.416	2.25E-24
TRBC2	2.00E-35	1.895	0.918	0.317	7.33E-31
CXCL13	3.85E-35	1.854	0.672	0.149	1.41E-30
TXNDC5	2.26E-29	1.836	0.984	0.560	8.28E-25
POU2AF1	2.01E-48	1.828	0.836	0.190	7.34E-44
CD79A	1.08E-44	1.799	0.852	0.205	3.95E-40
RGS1	1.38E-27	1.798	0.836	0.308	5.05E-23
SRGN	1.01E-25	1.777	0.934	0.524	3.70E-21
LYZ	2.09E-23	1.752	0.984	0.612	7.65E-19
SMAP2	8.53E-35	1.741	0.951	0.382	3.12E-30
TENT5C	2.46E-41	1.681	0.754	0.169	9.00E-37
LUM	7.23E-15	1.668	0.918	0.708	2.65E-10
SEC11C	4.87E-27	1.659	0.902	0.426	1.78E-22
CCL18	5.37E-15	1.654	0.754	0.362	1.97E-10
SLAMF7	1.16E-28	1.622	0.705	0.218	4.23E-24
ARHGDIB	1.73E-23	1.620	0.885	0.466	6.33E-19
PTGDS	3.26E-14	1.620	0.689	0.320	1.19E-09
TMSB4X	2.35E-21	1.610	1	0.951	8.59E-17
HLA-DRA	1.98E-21	1.603	1	0.868	7.26E-17
CYBA	6.10E-21	1.598	1	0.749	2.23E-16
FUCA1	4.33E-29	1.589	0.754	0.223	1.59E-24
CORO1A	1.68E-26	1.589	0.902	0.382	6.16E-22
CD53	2.44E-30	1.549	0.820	0.261	8.93E-26
IGFBP3	7.90E-12	1.531	0.689	0.392	2.89E-07
CD74	6.51E-21	1.521	1	0.902	2.38E-16

Table S6 | Representative Metabolites in OSF-derived OSCC Detected by AFADESI-MSI

Metabolites	mode	Formula	Adduct	Monoisotopic Molecular Weight	Theoretical m/z	Measured m/z	Relative Error (ppm)
Glucose	neg	C6H12O6	+Cl	180.0634	215.0328	215.0317	5.0710
Gluconic acid	neg	C6H12O7	-H	196.0583	195.0510	195.0511	-0.3759
Malic acid	neg	C4H6O5	-H	134.0215	133.0142	133.0143	-0.3966
Cholesterol sulfate	neg	C27H46O4S	-H	466.3117	465.3044	465.3044	0.0099
Palmitoleic acid	neg	C16H30O2	-H	254.2246	253.2173	253.2174	-0.3785
Linoleic acid	neg	C18H32O2	-H	280.2402	279.2330	279.2330	-0.1640
PS(38:4)	neg	C44H78NO10P	-H	811.5363	810.5291	810.5292	-0.1750
Aspartate	neg	C4H7NO4	-H	133.0375	132.0302	132.0303	-0.5176

Glutamine	neg	C5H10N2O3	-H	146.0691	145.0619	145.0619	-0.2334
Glutamate	neg	C5H9NO4	-H	147.0532	146.0459	146.0459	-0.1251
Argininosuccinic acid	neg	C10H18N4O6	-H2O-H	290.1226	271.1042	271.1030	4.5628
Adenine	neg	C5H5N5	-H	135.0545	134.0472	134.0460	9.0948
AMP	neg	C10H14N5O7P	-H	347.0631	346.0558	346.0558	-0.0338
ADP	neg	C10H15N5O10P2	-H	427.0294	426.0221	426.0222	-0.1674
HCY	neg	C4H9NO2S	+FA-H	135.0354	180.0336	180.0326	5.5472
PE(28:1)	pos	C33H64NO8P	+H	633.4370	634.4442	634.4442	0.0490
PC(38:7)	pos	C46H78NO8P	+H	803.5465	804.5538	804.5539	-0.1478
Citrulline	pos	C6H13N3O3	+Na	175.0957	198.0849	198.0850	-0.4591
Histamine	pos	C5H9N3	+H	111.0796	112.0869	112.0869	0.2084
Arginine	pos	C6H14N4O2	+H	174.1117	175.1190	175.1190	-0.2754
N-Methylputrescine	pos	C5H14N2	+H	102.1157	103.1230	103.1230	-0.2471
Spermidine	pos	C7H19N3	+H	145.1579	146.1652	146.1652	-0.1801
Spermine	pos	C10H26N4	+H	202.2157	203.2230	203.2230	0.1127
Putrescine	pos	C4H12N2	+H	88.1000	89.1073	89.1078	-5.3368
Ornithine	pos	C5H12N2O2	-H2O+H	132.0899	115.0877	115.0868	7.6993
N1-Acetylspermine	pos	C12H28N4O	-H2O+H	244.2263	227.2241	227.2231	4.4889
N1-Acetylspermidine	pos	C9H21N3O	+H	187.1685	188.1757	188.1758	-0.1150
Proline	pos	C5H9NO2	+H	115.0633	116.0706	116.0708	-1.6835
Glutamate-1-semialdehyde	pos	C5H9NO3	-H	131.0580	130.0505	130.0510	-3.8447
Methionine	pos	C5H11NO2S	+H	149.0510	150.0583	150.0582	0.6354
SAM	pos	C15H23N6O5S	M+	399.1451	399.1451	399.1433	4.4184
MTA	pos	C11H15N5O3S	+Na	297.0896	320.0788	320.0800	-3.8184
ATP	pos	C10H16N5O13P3	+Na	506.9957	529.9850	529.9851	-0.2020
L-Cystathionine	pos	C7H14N2O4S	+H	222.0674	223.0747	223.0747	0.0166

Table S7 | Primers for qRT-PCR

Primer names	Primer
FOSL1-Forward	5’-CAGGCGGAGACTGACAAACTG-3’
FOSL1-Reverse	5’-TCCTTCCGGGATTTTGCAGAT-3’
ANXA1-Forward	5’-CTAAGCGAAACAATGCACAGC-3’
ANXA1-Reverse	5’-CCTCCTCAAGGTGACCTGTAA-3’
COL17A1-Forward	5’-TCCACCCGATGGACAGAATTG-3’
COL17A1-Reverse	5’-GTAGGTGCCTGACACCGAC-3’
TCF4-Forward	5’-CAAGCACTGCCGACTACAATA-3’
TCF4-Reverse	5’-CCAGGCTGATTCATCCCACTG-3’
COL1A1-Forward	5’-GAGGGCCAAGACGAAGACATC-3’
COL1A1-Reverse	5’-CAGATCACGTCATCGCACAAC-3’
COL3A1-Forward	5’-TTGAAGGAGGATGTTCCCATCT-3’
COL3A1-Reverse	5’-ACAGACACATATTTGGCATGGTT-3’

Table S8 | List of Antibodies for Immunofluroscence and Western Blotting Experiments

Antibody	Catalog Number	Company
Anti-human Integrin Beta 1 Rabbit Polyclonal Antibody	12594-1-AP	Proteintech
Anti-human Granulin Rabbit Monoclonal Antibody	AWA10520	Abiowell
Anti-human Sortilin Rabbit Polyclonal Antibody	12369-1-AP	Proteintech
Anti-human CD20 Mouse Monoclonal Antibody	AWA00414	Abiowell
Anti-human COL17A1 Recombinant Rabbit Monoclonal Antibody	AWA10021	Abiowell
Anti-human COL3A1 Mouse Monoclonal Antibody	AWA00128	Abiowell
Anti-human COL1A1 Mouse Monoclonal Antibody	AWA00725	Abiowell

Anti-human FRA1 Recombinant Rabbite Monoclonal Antibody	AWA10022	Abiowell
Anti-human CD3 Recombinant Rabbit Monoclonal Antibody	AWA10523	Abiowell
Anti-human PLA2G2A Recombinant Rabbit Monoclonal Anitbody	AWA10131	Abiowell
Anti-human Annexin I Rabbit Polyclonal Antibody	AWA42508	Abiowell
Anti-human TCF4 Rabbit Polyclonal Antibody	22337-1-AP	Proteintech
Anti-human E-Cadherin Mouse Monoclonal Antibody	AWA01746	Abiowell
Anti-human Vimentin Mouse Monoclonal Antibody	AWA01597	Abiowell
